# Estimating the ecological drivers of insect abundance when detection is imperfect

**DOI:** 10.1111/1365-2656.70159

**Published:** 2025-10-22

**Authors:** Jens Ulrich, Risa D. Sargent

**Affiliations:** ^1^ Faculty of Land and Food Systems University of British Columbia Vancouver British Columbia Canada

**Keywords:** abundance estimation, binomial N‐mixture models, detection, GLMs/GLMMs, habitat restoration, insect sampling, mark–recapture, multinomial N‐mixture models

## Abstract

1. Biodiversity conservation hinges on a clear understanding of the ecological drivers of species abundance. In studies of insect abundance, researchers often estimate the effects of hypothesized drivers by applying generalized linear models and generalized linear mixed models (GLMs/GLMMs) to count data. However, a significant issue with conventional GLMs/GLMMs is that they cannot account for a failure to detect some individuals that are present (‘imperfect detection’), which can bias model estimates. To account for this, some researchers adopt hierarchical modelling approaches, including multinomial N‐mixture (multimix) models for mark–recapture data and binomial N‐mixture (binmix) models for repeated count data. Currently, we lack side‐by‐side comparisons to determine the ecological and study design conditions that require the use of these more cumbersome approaches to achieve accurate estimates.
2. We collected abundance data on wild bees in a study designed to compare unrestored to restored urban parks, which had more flowers and taller vegetation. We applied all three modelling approaches to these data, using either mark–recapture data (multimix approach) or ignoring whether individuals were marked and treating the data as traditional counts (binmix and GLMM approaches).
3. Our models indicated that capture rates for individual bees were below ~5%. The multimix mark–recapture model found that bees were ~1.6‐fold more likely to be detected in restored habitats. A GLMM, which did not account for detection bias, overestimated the effects of restoration on bee abundance. Using simulation, we found that multimix mark–recapture models had the highest accuracy and precision for estimating an abundance driver, including when individuals have the potential to move in/out of sampling areas; however, we also found that increasing baseline detection rates minimized the impacts of detection bias on GLMM estimates.
4. Our results emphasize that environmental factors can influence our ability to detect insects in field studies, and that these factors may be confounded with the experimental design. We recommend that studies planning to apply GLMs/GLMMs to count data prioritize methods that maximize detection over other aspects of study design such as the number of sites. Together, our results provide needed guidance on how to design and implement studies that accurately quantify the ecological drivers of insect abundance.

## Graphical abstract

Using a field‐based mark–recapture approach, the authors demonstrate that habitat quality can simultaneously influence insect abundance and detection. Simulation also showed that prioritizing increased sampling intensity over adding more study sites can improve accuracy of standard modelling approaches including GLMs or GLMMs, which do not account for detection confounders.

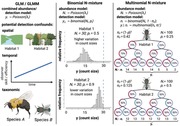

## 1 INTRODUCTION

Insects provide critical ecosystem services including pollination, decomposition and biological control (Schowalter et al., 2018). At the same time, many insects are facing widespread declines (Sánchez‐Bayo & Wyckhuys, 2019). In order to design evidence‐based strategies for mitigating or reversing insect declines, we require a clear understanding of the environmental pressures that underlie species abundance patterns (Wagner et al., 2021). Quantifying the ecological drivers of species abundance allows us to assess population trends, identify population threats and/or evaluate the success of management interventions (Blaauw & Isaacs, 2014; Schultz & Hammond, 2003; Waltz & Covington, 2004).

**FIGURE 1:**
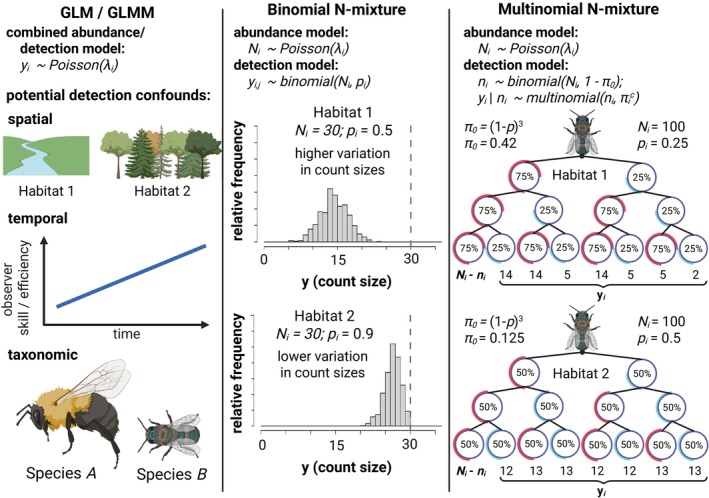
Comparison of three methods for estimating insect abundance. Abundance GLMs/GLMMs (column 1) treat count data ($y_{i}$) as a stochastic outcome with a mean value ($\lambda_{i}$) that may vary among spatial units, time periods or species. GLMMs are unable to disentangle the effects of these predictors on abundance from their potential effects on detection rate, for example, habitat impacts detectability; observers improve detection skills over time; or larger and more colourful species are detected at a higher rate than smaller and more dull‐coloured species. Binmix models (column 2) leverage the variation in counts ($y_{i}$) across repeated surveys (j) to simultaneously estimate detection rate (p) and total abundance ($N_{i}—$represented as dashed line). Many count surveys with low variation in Habitat 2 is consistent with higher detection rate (counts nearly approximate $N_{i}$). Higher variation among counts in Habitat 1 is consistent with lower detection rate (counts heavily underestimate $N_{i}$). Multimix models for mark–recapture data (column 3) aim to estimate the number of individuals in a population never detected ($N_{i}-n_{i}$) by classifying $y_{i}$ as the number of individuals in each observable encounter history. The total number of individuals detected one or more times ($n_{i}$) is dependent on an unobserved total population size ($N_{i}$) and the probability of an individual having any of the observable detection histories (1 – *π*_0_). In a study with three repeated surveys, the probability of never being captured ($\pi_{0}$) is equal to (1 − *p*)^3^. Detection histories can be tracked by placing marks on individual insects. Detection histories that skew towards infrequent observation in Habitat 1 suggest that there is a high probability of failing to detect individuals across all repeated surveys, that is, that $\pi_{0}$ is greater in Habitat 1 versus Habitat 2 and, correspondingly, that $n_{i}$ is likely a smaller proportion of $N_{i}$. Figure created in https://BioRender.com.

Accurately estimating the drivers of abundance is challenging because abundance itself is difficult to measure in natural environments. For insects, abundance data are often collected as counts from nets, traps or visual assessments (McCravy, 2018). Because many insect species are small, elusive and/or mobile, these counts undoubtedly overlook some number of individuals (Portman et al., 2020; Taron & Ries, 2015). To quantify the ecological drivers of abundance, researchers typically apply generalized linear models and generalized linear mixed models (GLMs/GLMMs) to these imperfect counts (Bolker et al., 2009). Crucially, GLMs/GLMMs rely on the assumption that the relationship between imperfectly detected counts and the true abundance of insects is constant across space, time and species. Systematic variation in the ability to detect individuals causes variation in this relationship and, consequently, has the potential to bias GLM/GLMM estimates of abundance drivers (Figure 1) (Williams et al., 2002). A concentration of food resources and/or habitat complexity could impact the ability to capture insects with nets or traps (Portman et al., 2020). For example, in a study of wild bees and flies, a higher density of flower resources on the day of a field survey improved the ability to detect insect species that were present (Ulrich & Sargent, 2025b). These effects of the environment on detection could confound GLM/GLMM estimates for abundance (Kery & Royle, 2020).

Mark–recapture is an alternative approach that estimates and accounts for imperfect detection. Essentially, researchers capture as many insects as possible within a prescribed location and time period. Researchers mark captured individuals with identifiable labels or markers, release them and later return and resample the population, recording the number of newly captured and recaptured (previously marked) individuals (Williams et al., 2002). Information about the probability of recapturing marked individuals across successive sampling events allows researchers to quantify and account for the detection rate while estimating abundance (Figure 1) (Williams et al., 2002). For decades, researchers have estimated abundance using mark–recapture (Lincoln, 1930), including for insects such as bees and butterflies (Briggs et al., 2022; Haddad et al., 2008).

Building on classic mark–recapture approaches for estimating abundance, hierarchical multinomial N‐mixture models (hereafter, ‘multimix’ models) allow researchers to pool mark–recapture data across multiple sites, treating site‐specific detection histories as the outcome of a stochastic process that is informed by the data obtained from all sites (Kery & Royle, 2010). Importantly, this approach allows researchers to estimate detection and abundance for sites with low or no recaptures. Compared to classic mark–recapture, sharing information about recapture rates across sites also increases estimate precision. In addition, this approach allows researchers to directly estimate potential predictors of abundance and detection such as site habitat quality. Furthermore, these models can be extended to estimate detection and abundance for communities of species simultaneously, rather than for single species independently (Kery & Royle, 2010). While mark–recapture methods are considered the most rigorous way to estimate drivers of insect abundance, they also have significant limitations. Collecting mark–recapture data can be difficult and time‐consuming (Taron & Ries, 2015). Additionally, placing marks could harm insects (Morton, 1982). Furthermore, multimix models for mark–recapture can require substantial coding experience and computational resources (Kery & Royle, 2020). Therefore, it is important to clarify the ecological and study design conditions that require the use of this more cumbersome method.

Binomial N‐mixture models (‘binmix’ models) are a third approach for estimating drivers of abundance. Binmix models are attractive because they can account for imperfect detection while being applied to the same count data used for GLMs/GLMMs (Madsen & Royle, 2023; Royle, 2004). Binmix models account for imperfect detection by leveraging information about the variation in the size of counts collected across repeated surveys (Figure 1) (Royle, 2004). Binmix models are increasingly utilized for insect abundance studies (Mimnagh et al., 2023). For example, researchers recently used them to determine the effects of urban land use on mosquito abundance, the impacts of flower resource density on wild bee abundance, the effects of seasonality on butterfly abundance, and to compare sampling methods for grasshoppers and crickets (Manica et al., 2019; Mccune et al., 2020; Nodari et al., 2025; Riva et al., 2020).

While binmix approaches provide an easier way to account for detection biases, they are not without controversy (Link et al., 2018). Both binmix and multimix mark–recapture models assume that populations are ‘closed’, that is, that population size is stable across repeated survey events (Kery & Royle, 2020). Movement of individuals in and out of sampling areas or substantial births/deaths across repeated surveys would violate the assumption of population closure (Kery & Royle, 2020). Given the potential for closure violations for many ecological systems, some researchers interpret estimates of abundance as estimates of the number of individuals from a wider area that spend some portion of time within the sampling area during the window of repeated surveys, that is, a ‘superpopulation’ (Kery & Royle, 2020). However, simulation studies show that even small variation in the number of individuals contained within a sampling area across repeated surveys can severely bias binmix estimates of the size of the superpopulation (Duarte et al., 2018; Link et al., 2018). Currently, there is limited information on how binmix models versus multimix models are impacted by violations of population closure assumptions, such as would be caused by movement or mortality.

Sensitivity of binmix models to modelling choices raises further concerns about their usage. Model misspecification, for example, not accounting for overdispersion in abundance or heterogeneity in individual detection rates, can impact estimation (Knape et al., 2018; Link et al., 2018). Monroe et al. (2019) also showed that trend estimates in population size are biased when covariate effects are confounded with changes in detection rate. Nonetheless, some studies have shown that binmix models provide good comparisons of relative abundance across sites under certain conditions, for example, when violations such as overdispersion or lack of closure do not covary with ecological predictors of abundance (Fogarty & Fleishman, 2021; Goldstein & de Valpine, 2022). Because of the potential sensitivity of binmix models to modelling assumptions, several studies recommend using mark–recapture methods to estimate abundance (Barker et al., 2018; Link et al., 2018). Currently, however, we lack direct comparisons of how population closure violations impact the ability for binmix and multimix models to estimate effect sizes for environmental covariates that are hypothesized to drive variation in abundance across sites.

Multimix mark–recapture models, binmix models and GLMs/GLMMs each have trade‐offs in terms of their required effort and performance. Researchers will want to implement the simplest approach that accurately and precisely estimates drivers of insect abundance. To test the performance of each approach under specific conditions, we conducted a field study to assess the effects of an environmental covariate (urban park restoration) on the detectability and abundance of wild bees. Here, we use ‘abundance’ to mean the number of individual bees that have some distributional overlap with a sampling area during the temporal window of sampling. We used net collection methods to obtain mark–recapture detection histories for wild bee species in urban parks with or without restorations. Restored parks had taller, unmowed vegetation and more floral resources (Ulrich & Sargent, 2025b). While a number of authors have argued that habitats with more flowers or taller vegetation impact bee detection rates, we currently lack studies that quantify these effects (Portman et al., 2020). We expected that restored parks would be associated with higher wild bee detection rates. In restored parks, bees foraging in taller vegetation would be closer to eye level and therefore could be easier to see and capture (McNeil et al., 2019). Furthermore, because floral resource density is associated with more sedentary insect foraging behaviour, greater floral resources in restored parks could also make bees easier to collect (Heinrich, 1979). We fit a multimix model to our mark–recapture data to quantify the effect of restoration on bee abundance and detectability. Alternatively, we ignored the individual marks and treated our data as if they were simply repeated counts collected for a GLMM or binmix model. This allowed us to determine how ignoring potential detection bias or using a less robust detection model influenced our estimates of a hypothesized driver of insect abundance.

Next, using simulation, we investigated factors that could influence the accuracy and precision of GLM/GLMM and binmix approaches compared to multimix mark–recapture. First, we examined whether changes in important aspects of ecological and study design conditions—baseline detection rate and/or number of study sites—impact the ability of these models to estimate the effect of an abundance driver that has a confounding effect on detection. In addition, we tested how violations of the assumption of population closure impacted the ability of binmix and multimix models to estimate an abundance driver. In combination, our results provide needed information on how to better design and conduct unbiased insect abundance field studies.

## 2 MATERIALS AND METHODS

### 2.1 Field study design and data collection

We collected wild bee abundance data from five urban parks in Vancouver, Canada, that had received habitat restoration amendments in 2020 and from five similar parks without restorations (hereafter, ‘control’ parks) (Table S1). In restored parks, a section of conventional turfgrass was excluded from regular mowing during April–September. In control parks, turfgrass was mowed approximately once every 2 weeks. Restored parks were also seeded with a flower mix. A separate study found that restoration of these same parks was strongly associated with increased plant species richness and marginally associated with increased floral abundance (Ulrich & Sargent, 2025b). Although we did not measure vegetation complexity, vegetation was approximately knee‐height (~50–75 cm) in restored parks compared to approximately ankle height in control parks (Figure S1). We collected abundance data in 2022 and again in 2023. Site centroids were separated by >1 km, approximately the maximum foraging distance for many bee species (Greenleaf et al., 2007). This allowed us to maintain spatial independence of the abundance for each site, despite the potential for individuals to move in and out of the site sampling areas.

We targeted our surveys on eight wild bee species from four different families: *Agapostemon texanus* (Halictidae), *Andrena prunorum* (Andrenidae), *Anthidium oblongatum* (Megachilidae), *Bombus flavifrons* (Apidae), *Bombus mixtus* (Apidae), *Halictus rubicundus* (Halictidae), *Megachile* spp. (not including the non‐native alfalfa leafcutter, *Megachile rotundata*) (Megachilidae) and *Melissodes microstictus* (Apidae) (Figure S2). We chose these species/species groups because we could easily identify them in the field. Identification of many other species or species groups requires lethal sampling. However, we could not kill bees and still carry out mark–recapture. Additionally, in our system, these species were common enough that we could estimate the timing of peak abundance from a previous study (Ulrich & Sargent, 2025b). We collected data during two different sampling periods. In early June, we conducted three repeated surveys for species with ‘early’ phenology: *Agapostemon texanus*, *B*. flavifrons and *B. mixtus*. Then, in mid‐July, we conducted the repeated surveys for five species with ‘late’ phenology: *Andrena prunorum*, *A. oblongatum*, *H. rubicundus*, *Megachile* spp. and *M. microstictus*. We conducted repeated surveys within a rapid timescale, conducting the first and last repeated surveys at each site within a maximum of 8 days. Flight periods for individual wild bees typically last at least several weeks (Larsson & Franzén, 2008), wherein individual bees tend to limit foraging activity within a few hundred metres of their activity centre (Ogilvie & Thomson, 2016). We only collected female bees. *Andrena prunorum* was only surveyed in 2023. Our approach does not account for potential heterogeneity in abundance and detection responses among the native *Megachile* species that we grouped together.

We conducted the abundance surveys in one‐hectare sampling areas. The sampling areas focused on a restored vegetation area in the restored parks and an area of conventionally managed turfgrass in control parks. We collected bees using a sweep net method, walking a spiral transect through the survey area and netting all individuals thought to be one of our focal species (McCravy, 2018). We repeated the spiral transect until the survey time ended. Surveys lasted 45 min, with the timer paused for insect handling. Netted insects were transferred into individual vials and placed on ice until the completion of the survey. Placing insects on ice reduced movement and activity, allowing us to confirm species identifications and apply individual marks without causing further distress. We applied marks by placing chilled bees in a vial with a plunger on the one end and a mesh covering on the other end (Briggs et al., 2022). Pressing bees against the mesh to inhibit movement, we painted a colour‐coded mark on the back of the thorax (Molotow brand paint pen, 2.0 mm round tip) (Figure S3). Handling and/or marking insects could increase mortality (Gall, 1984), although we observed bees resume their active foraging behaviour within a few minutes of being released. To implement the GLMM and binmix model approaches, we recorded the total number of captured individuals, ignoring whether individuals were marked. To limit observer biases, all surveys were conducted by the same researcher, only during full sun conditions between 10:00 AM and 4:00 PM.

The Vancouver Parks Board granted licences and permits to conduct fieldwork. This study did not require ethical approval.

### 2.2 Statistical analyses

To estimate the effects of restoration on insect abundance, we developed custom models written in the probabilistic programming language Stan (Stan Development Team, 2023b), implemented in R Version 4.2.2 (R Core Team, 2022) with *rstan* (Stan Development Team, 2023a). These models use Bayesian inference to estimate distributions of parameter values that are conditioned on our data and prior probability distributions.

For the GLMM model, we specified counts of $y_{i}$ individuals from each sampling event as the outcome of a negative binomial distribution with a mean $\lambda_{i}$ and dispersion ϕ (Equation 1). We used negative binomial distributions for the GLMM (and the binmix and multimix models) because Poisson models showed a clear lack of fit (Figure S4). Using a log‐link function, we modelled variation in $\lambda_{i}$ (Equation 2) as a function of a species‐specific random intercept effect ($\alpha_{1}$), a site‐specific random intercept effect ($\alpha_{2}$), a fixed effect of year ($\alpha_{3}$) and a species‐specific random slope effect of restoration ($\alpha_{4}$), with restoration treated as a categorical variable.

(1) $$y_{i}\sim \text{negative binomial}\left(\lambda_{i},\phi \right);$$

(2) $$\log\left(\lambda_{i}\right)=\alpha_{1}\left[\text{species}\right]+\alpha_{2}\left[\text{site}\right]+\alpha_{3}\left[\text{year}\right]+\alpha_{4}\left[\text{species}\right]\times \text{restoration}\left[i\right].$$

We assumed that species‐specific random intercepts and effects of restoration were normally distributed, $\alpha_{1}\left[\text{species}\right]\sim \text{normal}\left(\mu_{\alpha_{1}},\sigma_{\alpha_{1}}\right)$ and $\alpha_{4}\left[\text{species}\right]\sim \text{normal}\left(\mu_{\alpha_{4}},\sigma_{\alpha_{4}}\right)$, where $\mu_{\alpha_{1}}$ and $\mu_{\alpha_{4}}$ represent the mean abundance intercept and the mean effect of restoration on abundance across all bee species.

For our binmix model, we assumed that there was an imperfectly observed latent abundance, $N_{i}$, for each site/species/year combination, i. We considered $N_{i}$ to be the outcome of a negative binomial distribution with a mean $\lambda_{i}$ and dispersion ϕ (Equation 3). We treated the number of successfully detected individuals, $y_{i,j}$, on each sampling event, j, as the outcome of a binomial trial with size $N_{i}$ and a success rate, $p_{i}$. This formulation allowed us to differentiate the effects of predictors on abundance from their effects on detection (Kery, 2017). We described variation in $\lambda_{i}$ as in Equation (2). Using a logit‐link function, we modelled variation in $p_{i}$ as function of a species‐specific random intercept effect ($\beta_{1}$), a site‐specific random intercept effect ($\beta_{2}$), an effect of year ($\beta_{3}$) and a species‐specific random slope effect of restoration ($\beta_{4}$) (Equation 5).

(3) $$N_{i}\sim \text{negative binomial}\left(\lambda_{i},\phi \right);$$

(4) $$y_{i,j}\sim \text{binomial}\left(N_{i},p_{i}\right);$$

(5) $$\text{logit}\left(p_{i}\right)=\beta_{1}\left[\text{species}\right]+\beta_{2}\left[\text{site}\right]+\beta_{3}\left[\text{year}\right]+\beta_{4}\left[\text{species}\right]\times \text{restoration}\left[i\right].$$

We assumed that species‐specific random intercepts and effects of restoration were normally distributed, $\beta_{1}\left[\text{species}\right]\sim \text{normal}\left(\mu_{\beta_{1}},\sigma_{\beta_{1}}\right)$ and $\beta_{4}\left[\text{species}\right]\sim \text{normal}\left(\mu_{\beta_{4}},\sigma_{\beta_{4}}\right)$, where $\mu_{\beta_{1}}$ and $\mu_{\beta_{4}}$ represent the mean abundance intercept and the mean effect of restoration on abundance across all bee species.

Stan does not support direct sampling of latent discrete variables, that is, where the likelihood of the data is estimated with respect to a sampled value of $N_{i}$ (Stan Development Team, 2023b). Instead, following standard approaches, we calculated the marginal likelihood of the data across a range of $N_{i}$, from a lower bound of the largest count observed for a species at a site across all three surveys to an upper bound of a large finite value $K_{i}$ (Hocking et al., 2021; Royle, 2004). K should be large enough that values of N approaching K do not significantly contribute to the likelihood (Royle, 2004). For the field study, we used a value of $K_{i}=\left(\text{largest observed count}+5\right)\times 12$. Our model estimation proceeds by proposing new values for the abundance and detection parameters and then evaluating whether the likelihood of the data across all possible values of $N_{i}$ improves. Other programmes employ default values of the maximum possible abundance, for example, $K_{i}=\left(\text{largest observed count}\right)+100$ for the popular R program unmarked (Fiske & Chandler, 2011). Using custom models, we were able to increase K to higher limits, which allowed us to evaluate the sensitivity of our estimates to the choice of K to ensure that stable results were obtained (Kery, 2017).

Finally, for the multimix approach, we specified a three‐level model with a multinomial observation process for detection histories (Kery & Royle, 2010). Where 0 represents non‐detection and 1 represents detection, the possible observable detection histories for three repeat surveys are 111, 110, 101, 100, 011, 010 and 001. The eighth possible history, 000, represents unobserved individuals (Royle et al., 2007). The outcome variable $y_{i}$ was defined as a vector with each element representing the number of individuals in each of the seven observable detection history categories (Figure 1). The likelihood of the observed detection histories is specified as the outcome of a multinomial trial with some number of individuals observed one or more times ($n_{i}$), with a vector of probabilities for each observable history ($\pi_{i}^{c}$) (Equation 6). To estimate the remaining number of individuals at the site, we treated the number of observed individuals ($n_{i}$) as the outcome of a binomial trial that depends on the size of the total population ($N_{i}$) and the probability of not being observed ($\pi_{i}^{0}$, i.e. probability of a detection history of 000) (Equation 7):

(6) $$y_{i}\sim \text{multinomial}\left(n_{i},\pi_{i}^{c}\right);$$

(7) $$n_{i}\sim \text{binomial}\left(N_{i},1-\pi_{i}^{0}\right).$$

As with the binmix model, the population size $N_{i}$ was modelled as the outcome of a negative binomial distribution (Equation 3) with a dispersion term and a linear predictor for the mean (Equation 2). We bounded $N_{i}$ within the range $K_{i}=\left(\left(n_{i}+5\right)\times 12\right)$. Heterogeneity in the detection rate was modelled using a linear predictor for $p_{i}$ (Equation 5), but where $p_{i}$ is estimated by defining its relation to $\pi_{i}^{1:8}$. For example, where probability of detection history 111 is $\pi_{i}^{1}=p_{i}\times p_{i}\times p_{i}$, or probability of detection history 110 is $\pi_{i}^{2}=p_{i}\times p_{i}\times \left(1-p_{i}\right)$ (Figure 1). By tracking individual level detection histories, our multimix model estimates for $p_{i}$ combine the probability that an individual is alive and actively foraging in the sampling area at the time of a sampling event along with the probability that we see and capture the individual with a net.

We employed best practices for model development and fitting by (1) using weakly informative priors to discourage unrealistic parameter values (Tables S2–S4); (2) confirming sufficient mixing of chains (Gelman‐Rubin *R*‐hat values <1.05), minimal within‐chain autocorrelation (effective sample size/steps >0.1) and no divergent transitions (Figures S5 and S6); (3) running our model for a length of 4000 iterations, discarding the first 2000; and (4) using posterior predictive checks to assess the fit of our models (Figure S4) (Gelman et al., 2020; Kery & Royle, 2020; Stan Development Team, 2023b).

### 2.3 Simulation study

We wrote a simulation program in R to generate species abundance and detection patterns. We simulated abundance from a Poisson distribution. We included species, site and year predictors for the mean of the Poisson generating process, as in Equation (2). Conditional on input abundance and detection rates, the programme then produces mark–recapture histories or summarizes the histories as counts. The simulation script allows the detection rate to vary by species, site and/or year, as in Equation (5).

We used this simulation to determine how particular aspects of study design and violations of the population closure assumption impact estimates of the effect of an ecological driver of abundance. To accomplish this goal, we fit each of the three models to data that were simulated under different study parameter scenarios. For all simulations, we specified half of the sites as ‘restored’ and the other half of sites as ‘control’. In these simulations, restoration represents a hypothesized ecological driver of insect abundance that a researcher may wish to quantify. The effect of the restoration parameter, $\mu_{\alpha_{4}}$ (Equation 2), was held constant at 1 (a positive effect).

First, we assessed how changes in baseline insect detection rates impacted model bias and precision. We simulated detections for 20 sites, 16 species and 2 years, with three repeated surveys per year. We varied the mean detection intercept ($\mu_{\beta_{1}}$) across values of −3, −2, −1 and 0 (on a logit‐scaled). On the probability scale, a value of 0 represents a 50% probability of an individual insect being detected during a given survey given that it is present, whereas a value of −3 represents a < 5% probability. For each level of detectability, we varied the effect of habitat restoration on detection rate ($\mu_{\beta_{4}}$) across values of −0.5, 0, 0.5 and 1 (logit‐scaled). A positive value corresponds to a scenario where restored sites increase detection rate. The abundance intercept was held constant at a value of 2 (log‐scaled). We fit all three of the models to 20 systems simulated under each scenario. We extracted 250 samples from the posterior distribution of model estimates for each model fit. To assess model accuracy, we calculated the bias of the estimates: the difference between each of the sampled effect size estimates and the true value of the effect of habitat on abundance ($\mu_{\alpha_{4}}$), that is, how far away the parameter estimate is from the true value of 1 that was used to simulate the data. We calculated model precision as the reciprocal of the standard deviation of all posterior samples from each simulation scenario. Higher precision reflects lower uncertainty in the posterior distributions. Next, we assessed how changes in the number of sites included in a study impacted model accuracy and precision. We simulated scenarios with 10, 20 or 30 sites. For each scenario, we again varied the effect of habitat restoration on detection rate. The detection intercept was held constant at −2 (logit‐scaled). All other parameters and study dimensions were held constant as described above.

Finally, we examined how violations of the population closure assumption impacted binmix and multimix estimates. Here, we still considered $N_{i}$ to be the outcome of a Poisson trial. However, we also simulated movement in and out of sites by allowing random variation in whether individuals were ‘available’ for detection: Each individual, k, from each superpopulation, $N_{i}$, was randomly available for detection on sampling event, j, at site *i* with some probability $\theta_{i}$: ${\text{availability}}_{i,j,k}\sim \text{binomial}\left(N_{i},\theta_{i}\right)$. We specified a linear predictor for $\theta_{i}$, with an availability intercept $\theta_{0}$ and an effect of habitat restoration on availability $\theta_{1}$. We varied $\theta_{0}$ across −1, 0 and 1; and $\theta_{1}$ across −1, 0 and 1 (logit‐scaled). A negative value of $\theta_{1}$ represents a scenario where restoration reduces the tendency for individuals to remain within the sampling area, whereas a positive value represents a positive effect on this tendency. A value of zero indicates a scenario where individuals may move in and out of the sampling area, but that restoration does not affect the probability of doing so. For these simulations, we held the abundance intercept at 3 (log‐scaled) and kept the baseline detection rate constant at −2 (logit‐scaled) with no effect of habitat restoration on detection. We used a larger abundance intercept for these simulations to reflect a larger number of individuals in the wider landscape surrounding a site that have some probability of entering the sampling area during each survey. All other parameters and study dimensions were held constant as described above, with the same approach used to quantify accuracy and precision.

## 3 RESULTS

**FIGURE 2:**
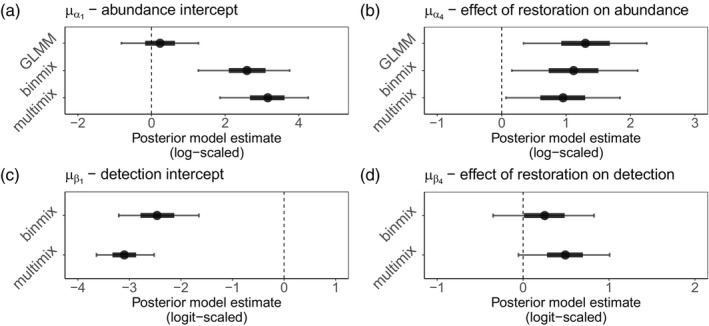
Field study estimates of wild bee abundance and detection. We estimated abundance intercepts (a) and community mean effect of restoration on abundance (b) with all three model types. We estimated detection rate intercepts (c) and community mean effect of restoration on detection (d) for binmix and multimix models, which are designed to estimate and account for imperfection detection. Mean estimates are displayed with filled circles. Thick and thin error bars represent 50% and 90% Bayesian credible intervals.

Using binmix and multimix models, we estimated wild bee detection rates in the field (Figure 2). With the multimix model, we estimated a lower baseline detection rate ($\mu_{\beta_{1}}$ mean = −3.17; 90% Bayesian credible interval (BCI) = [−3.66, −2.62]) than with the binmix model ($\mu_{\beta_{1}}$ mean = −2.47; 90% BCI = [−3.2, −1.65]). On a probability scale, the mean estimate of the multimix model equates to a 4.3% probability of detecting an individual bee during a survey of a control plot (90% BCI = [2.5%, 7.4%]). The multimix model estimated a positive effect of restoration on detection: $\mu_{\beta_{4}}$ mean = 0.56; 90% BCI = [0.07, 1.05]. On a probability scale, the mean estimate of the multimix model equates to a 6.8% probability (1.6 fold increase) of detecting individual bees during surveys of restored sites (90% BCI = [3.9%, 11.8%]). The binmix model tended to estimate a positive effect of restoration on detection rate, although the 90% BCI overlapped with zero: $\mu_{\beta_{4}}$ mean = 0.25; 90% BCI = [−0.34, 0.83].

We used all three model types to estimate the effect of habitat restoration on wild bee abundance (Figure 2). The GLMM estimated a positive effect of restoration on insect abundance: $\mu_{\alpha_{4}}$ mean = 1.30; 90% BCI = [0.34, 2.25]. Effect size estimates for the binmix model were smaller: $\mu_{\alpha_{4}}$ mean = 1.11; 90% BCI = [0.16, 2.11]. The multimix model also estimated a smaller magnitude of effect of restoration on wild bee abundance: $\mu_{\alpha_{4}}$ mean = 0.91; 90% BCI = [0.05, 1.85]. Combined with the abundance intercept estimates, the mean estimates from the multimix model equate to a 2.6‐fold increase in the relative number of individuals that are present for the average species: exp(3.16) = ~24 individuals in unrestored parks versus exp(3.16 + 0.91) = ~58 individuals in restored sites. In contrast, the mean estimate from the GLMM equates to a 3.7‐fold increase in the relative number of individuals that are present: exp(0.23) = ~1.3 individuals in unrestored parks versus exp(0.23 + 1.30) = ~4.6 individuals in restored sites (Figure S7). Therefore, with the GLMM compared to the multimix model, we estimated nearly a 50% additional increase in the relative population size of the average wild bee species in restored versus unrestored parks.

**FIGURE 3:**
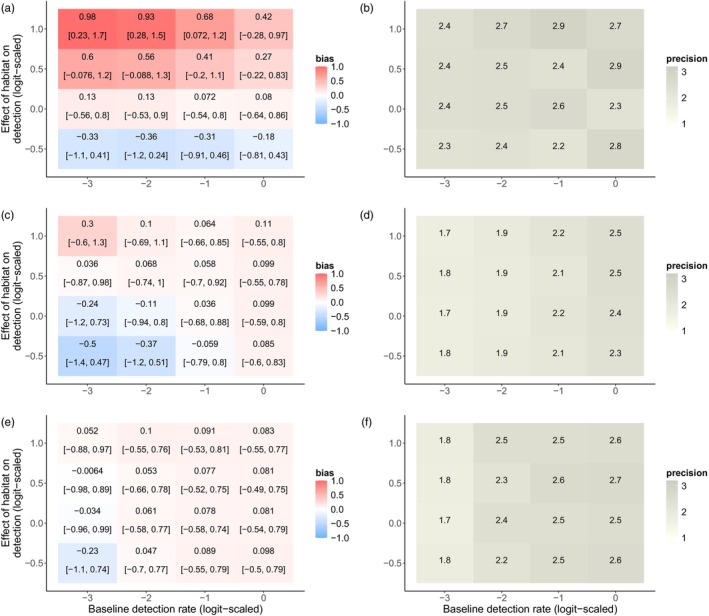
Effects of a simulated detection confounder on model accuracy under changes in baseline detection rate. We simulated abundance counts while simultaneously varying the effect of habitat on detection and the baseline detection rate (community mean detection intercept—$\mu_{\beta_{1}}$). We fit GLMMs (a, b), binmix (c, d) and multimix (e, f) models to the data and determined the accuracy and precision of the estimate for the effect of habitat on abundance. Positive bias (red) indicates a tendency to overestimate the effect of the hypothesized abundance driver; a negative bias (blue) indicates a tendency towards underestimation. The mean bias and 90% Bayesian credible intervals are displayed for each simulation scenario (a, c and e). Darker shades of grey indicate higher precision in the parameter estimates (b, d and f).

Our simulations determined how certain aspects of study design impact the ability of these models to estimate insect abundance drivers. First, we examined how changes in baseline detection rate and the effects of habitat restoration on the detection rate impact the accuracy and precision of estimates of the effect of habitat restoration on abundance. When the probability of detecting an insect was 50% (0 on the logit scale), GLMMs showed minimal bias even when habitat strongly increased detection rate (mean bias = 0.42; 90% BCI = [−0.28, 0.97]) (Figure 3). Lower detection rates increased bias: When the probability of detecting an insect was <5% (−3 on the logit scale), there was a clear positive bias when restoration increased detection rate (mean bias = 0.98; 90% BCI = [0.23, 1.7]). GLMM precision did not vary systematically with changes in baseline detection rate (Figure 3). Across all baseline detection rate levels, binmix and multimix models were not clearly biased by a confounding effect of habitat on detection (Figure 3). Increasing the baseline detection rate improved binmix and multimix precision (Figure 3).

**FIGURE 4:**
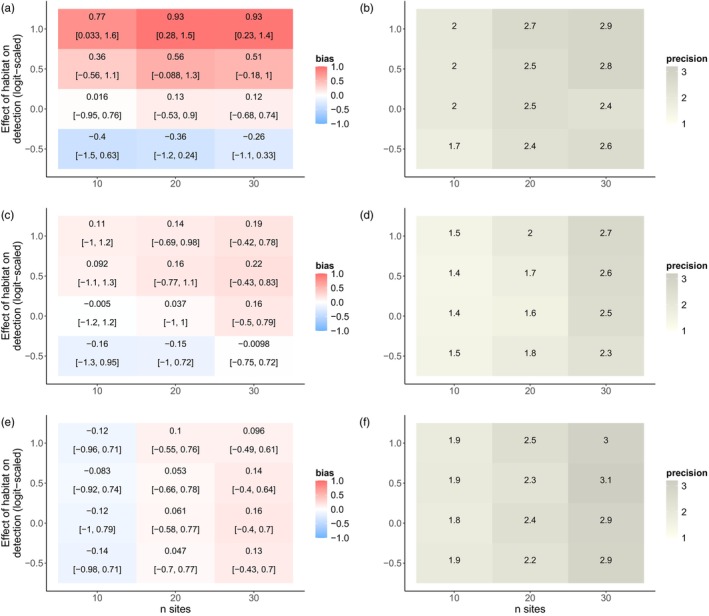
Effects of a simulated detection confounder on model accuracy under changes in the number of study sites sampled. We simulated abundance counts while simultaneously varying the effect of habitat on detection and the number of study sites that were sampled. We fit GLMMs (a, b), binmix (c, d) and multimix (e, f) models to the data and determined the accuracy and precision of the estimate for the effect of habitat on abundance. Positive bias (red) indicates a tendency to overestimate the effect of the hypothesized abundance driver; a negative bias (blue) indicates a tendency towards underestimation. The mean bias and 90% Bayesian credible intervals are displayed for each simulation scenario (a, c, and e). Darker shades of grey indicate higher precision in the parameter estimates (b, d and f).

Increasing the number of sites exacerbated GLMM biases (Figure 4), while simultaneously increasing estimate precision (Figure 4). When there was a strong positive effect of restoration on detection rate and only 10 study sites, GLMMs slightly overestimated the effect of restoration on abundance (mean bias = 0.77; 90% BCI = [0.03, 1.6]) with a relatively high degree of uncertainty (precision = 2) (Figure 4). Increasing the number of study sites to 30 amplified the bias (mean bias = 0.93; 90% BCI = 0.23, 1.4) while also increasing precision (precision = 2.9). Across variation in the number of sites, binmix and multimix models were not clearly biased by a confounding effect of habitat on detection (Figure 4). Increasing the number of study sites increased binmix and multimix precision (Figure 4). In general, multimix models were more precise than binmix models (Figures 3 and 4).

**FIGURE 5:**
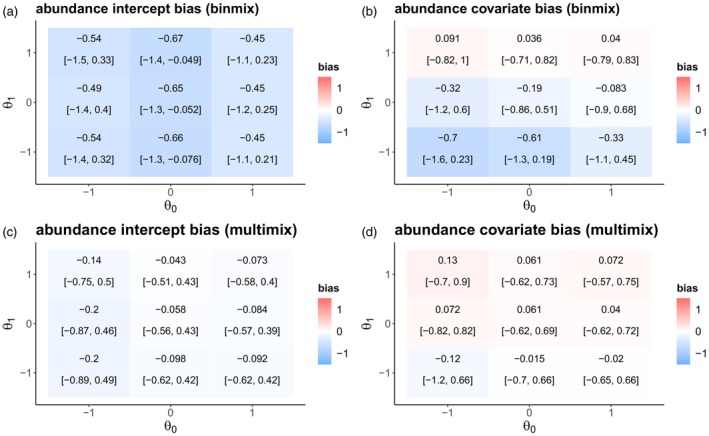
Robustness of binmix and multimx models to closure violations. We simulated variation in the probability that insects remain within a sampling area at the time of a survey event ($\theta_{0}$), and the degree to which a site covariate of habitat restoration impacts this probability ($\theta_{1}$). We recorded the bias for estimates of the abundance intercept and for the effect of a simulated abundance driver for both a binmix model (a, b) and for a multimix model (c, d). Positive bias (red) indicates a tendency to overestimate the target while a negative bias (blue) indicates a tendency towards underestimation. The mean bias and 90% Bayesian credible intervals are displayed for each simulation scenario.

Finally, we assessed the robustness of binmix and multimix models to violations of the closure assumption (Figure 5). Across all simulation scenarios, binmix models underestimated the abundance intercept term, whereas the multimix model did not. For both model types, abundance covariate estimates were robust to closure violations when the probability of movement into/out of sites was unrelated to site conditions ($\theta_{1}$ = 0). Binmix model estimates were more sensitive to systematic violations of the closure assumptions when the baseline probability of remaining in the site were low: When $\theta_{0}$ = −1 and $\theta_{1}$ = −1, the binmix model underestimated the effect of habitat restoration on abundance by an average of −0.70 (90% BCI = [−1.6, 0.23]). In contrast, across all simulation scenarios, the bias of the multimix model was ≤0.13.

## 4 DISCUSSION

To develop evidence‐based strategies to mitigate or reverse insect declines, we require the ability to estimate the effects of ecological factors on insect abundance patterns with accuracy and precision (Wagner et al., 2021). At the same time, insects are difficult to detect with standard ecological survey tools, complicating our ability to quantify the drivers of their abundance. From a sample of urban parks that contained restored or unrestored habitat, we collected wild bee abundance data that we could treat either as mark–recapture histories or as traditional counts. Supporting our hypothesis that more flowers and taller vegetation improve detection, a multinomial N‐mixture (multimix) model for the mark–recapture data estimated that bees were approximately 1.6‐fold more likely to be detected in sites with restored habitats compared to control sites. Our binomial N‐mixture (binmix) model for count data also suggested that individual bees were easier to detect in restored sites. Previous studies have shown that habitat quality impacts detection rate for other insect groups or for vertebrates such as birds (Manica et al., 2019; Royle et al., 2005). To our knowledge, this is the first quantitative evidence that habitat quality can impact our ability to measure wild bee abundance. A GLMM, which did not account for detection bias, overestimated the effects of restoration on bee abundance, estimating nearly 50% greater increases in abundance in restored versus control parks as compared to the multimix model. This highlights that confounding effects on detection can bias GLMM estimates of insect abundance drivers. As such, we should interpret effect sizes produced by GLMMs with caution when the environment has the potential to influence our ability to observe or capture insects. Finally, using simulation, we demonstrated that multimix mark–recapture models were the most robust and precise approach for estimating a hypothesized driver of abundance; however, we also found that increasing the baseline detection rate minimized the tendency for detection biases to impact GLMM estimates. This emphasizes that studies making use of GLMMs can increase their accuracy by prioritizing increased detection rates over other aspects of study design such as the number of sites. Together, our results provide needed guidance on how to design and implement studies that improve our understanding of how to conserve insects in a changing world.

While the GLMM approach exaggerated the size of the effect, all three models estimated a positive effect of restorations on wild bee abundance, consistent with the findings of a separate study of wild bee and hoverfly species occurrence in the same urban park system (Ulrich & Sargent, 2025b). More broadly, habitat amendments that increase floral resources tend to be associated with an increased abundance of insect pollinators (Hyjazie & Sargent, 2022). Pollinator habitat amendments, including reduced turfgrass mowing and wildflower seeding, are growing in popularity and offer a promising approach for supporting urban biodiversity conservation (Cloutier et al., 2024; Wolfin et al., 2023).

We found that habitats with more flowers and taller vegetation were associated with increased insect detectability. Recent studies have uncovered other factors that impact insect detection. For example, looking at urban mosquito populations, Manica et al. (2019) found that dense vegetation cover improved trap capture rates, aligning with preferences in the location of mosquito oviposition. Other studies have pointed to observer differences as sources of insect detection bias. For example, in a study of UK butterfly populations, Isaac et al. (2011) found that one field team had a higher butterfly detection rate than another, potentially due to differences in prior field experience among the teams. Another study found that larger and more brightly coloured butterfly species were easier to detect during transect walks compared to smaller and duller species, which could bias species abundance comparisons made by GLMMs (Kral‐O'Brien et al., 2020). Taken together, these results emphasize that an array of different environmental factors has the potential to confound GLMM estimates of insect abundance drivers. The use of random effects in our GLMMs handled non‐independence of sites and species in our data. GLMs, which do not account for the pseudoreplication of data in this type of study design, may perform even worse when there is a detection confound (Bolker et al., 2009).

Still, conventional GLMs/GLMMs are popular with ecologists and are likely to remain the standard methodology for insect population and community studies. Our simulation showed that increasing the baseline detection rate reduces the tendency for bias. Although bias was more evident when baseline detection rates were low (<25% on a probability scale), a confounding effect on detection rate did not consistently bias model estimates (90% BCI overlapping with zero) when baseline detection rates were held at ~50%. Conversely, adding more sites exacerbated GLMM bias while simultaneously increasing precision (i.e. adding more sites increased the confidence in an inaccurate conclusion). Under our simulation scenario, the expected abundance of the mean species in unrestored sites would be ~7 individuals, with an increase to ~20 individuals in restored sites. A bias of 0.5 would result in an expected abundance of 33 individuals in restored sites (overestimating increases in population size by ~100%), while a bias of 1 would result in an expected abundance of 55 individuals (overestimating increases in population size by ~270%) (Figure S7). This highlights that even small changes in parameter bias can have strong implications for our understanding of management impacts. If researchers planning to employ GLMMs are concerned about a site covariate impacting detection, our results indicate that study design should prioritize increased detection rate over site replication. Increasing the detection rate could be achieved by improved sampling intensity: for example, by increasing the duration of surveys, by increasing the number and/or expertise of surveyors or by increasing the number of traps deployed per sampling location (McCravy, 2018).

Across our field sites, the mark–recapture model estimated bee detection rates that ranged between 2% and 12%. These estimates are consistent with those provided by other mark–recapture bee studies; for example, a study of bees in North Carolina forests estimated capture probabilities ranging between 4% and 52% (Briggs et al., 2022). Detection rates may be even higher for some insects, such as Iolas blue butterflies (*Iolana iolas*), where researchers reported an 86% detection rate (Heer et al., 2013). Conversely, detection rates for other insects might be lower; researchers collecting mosquitos with passive traps reported a detection probability of <1% (Manica et al., 2019). Our results about the relationship between baseline detection rate and GLMM bias suggest that detection models or study design controls are especially important for insect taxa that are more difficult to detect.

Our simulations confirmed that even minor violations of the closure assumption cause severe bias for binmix estimates of total population size (Link et al., 2018). We found that binmix estimates of a hypothesized driver of abundance were only inaccurate when closure violations were systematic. Specifically, estimates of the simulated abundance driver were biased when closure violations were rampant (i.e. baseline probability of being within a sampling plot at the time of a survey were less than 50%), and simultaneously when there was a confounding effect of habitat on the degree of closure violations (i.e. when $\theta_{1}$ does not equal zero). Because it may be difficult to obtain a priori knowledge on whether the environment affects the rate of closure violations, more rigorous multimix mark–recapture models should be applied over binmix models in situations where populations are likely highly dynamic.

High mobility or rapid mortality makes populations more dynamic and increases the chance of population closure violations. For our study, we surveyed 1‐hectare plots. Although they can move further (Greenleaf et al., 2007), as central place foragers, wild bees tend to exhibit strong site fidelity within a few hundred metres (Ogilvie & Thomson, 2016). This means that individual bees likely remained within the 1‐hectare sampling areas across repeated surveys. Insects with larger daily movements and no fixed nest position may be less likely to satisfy the assumption of high closure. For example, the chequered skipper butterfly, *Carterocephalus palaemon*, can move more than a kilometre in a single day (Wildman et al., 2024). In addition, unlike bees, butterflies do not maintain fixed nest locations and may move among sampling areas (Ricketts, 2001). Therefore, butterflies may be less suitable for binmix approaches. Habitat edges such as roads or hedges impact insect movement (Markovits et al., 2025; Wratten et al., 2003). To minimize systematic closure violations caused by insect movement, researchers using binmix models should standardize the presence of discrete site borders such as roads. Repeated surveys that are conducted more rapidly relative to insect lifespan would also improve the robustness of binmix models. The activity periods for wild bees typically last several weeks (Larsson & Franzén, 2008), a reasonably broad window for our 8‐day repeated survey windows. Conducting repeated surveys within a single day could help limit closure violations for insects with shorter lifespans (Nowicki et al., 2008). Overall, binmix models are most appropriate when mobility is limited and individuals are longer lived. For example, where they were used for stag beetles (Lucanidae), which have months‐long activity periods wherein they tend to move less than a few dozen metres per day (Della Rocca et al., 2020).

In our simulation study, multimix models for mark–recapture data consistently provided the most accurate estimates of abundance drivers. Multimix models also provided more precise estimates of a hypothesized abundance driver, in agreement with other studies comparing estimates for total abundance (Bötsch et al., 2020). As such, multimix model approaches allow researchers to leverage maximum information from a limited sample size. This is especially important when a focal species is rare, occurs at a limited number of sites or where site survey permitting is a limiting factor. Relative to other biological groups, insects may be more conducive to mark–recapture approaches. During sampling, insects were easily placed on ice to induce calm behaviour and temporary sedation during sampling. This contrasts with the more intensive handling processes for vertebrates such as birds, which can cause distress and increased mortality (Putman, 1995). Furthermore, the smaller ranges of many insect species make repeat surveys and resighting more logistically feasible compared to wide‐ranging animals such as large mammals (Garshelis, 1992).

Multimix models and related model types can be applied to data generated using sampling protocols other than mark–recapture. Alternative protocols include removal sampling, multiple observer recording, territory tracking or distance sampling (Kery & Royle, 2020). Distance sampling appears promising for insect studies and has already been applied to studies of bumble bees and butterflies (Kral‐O'Brien et al., 2020; McNeil et al., 2019). With this approach, a researcher typically walks a transect through a sampling area, recording the distance between the observer and detected insects. Researchers then infer the detection probability from the observed pattern of distances. The distance sampling method is attractive because it does not require that individuals be marked, it does not require repeated surveys, and it does not require defining discrete sampling area boundaries (Kery & Royle, 2020; Taron & Ries, 2015).

In this study, we demonstrate that habitat restoration can simultaneously influence insect abundance and detection. Given their robustness and precision, we recommend using multimix approaches to account for detection biases, especially when insect mobility exceeds the spatial area of sampling or when insect life spans are shorter than the time period between repeated survey events. At the same time, multimix mark–recapture approaches are challenging to implement. New approaches such as photographic or genetic mark–recapture could make these approaches less cumbersome (Wildman et al., 2024). Integrated modelling approaches, which allow researchers to combine powerful mark–recapture data from a subset of sites or species with simple count data from a larger number of sampling units, could also prove useful (Jarrett et al., 2022). We anticipate that GLM and GLMM approaches will remain common for studies of insect abundance. As such, we recommend designing studies that minimize detection bias, which can be achieved by prioritizing increased sampling intensity per site over adding additional study sites that are poorly sampled. By accounting for detection biases or by minimizing detection biases through study design, researchers can provide the accurate and precise evidence needed to make recommendations for mitigating insect declines.

## AUTHOR CONTRIBUTIONS

Jens Ulrich and Risa D. Sargent conceived the ideas and designed the methodology; Jens Ulrich collected and analysed the data and led the writing of the manuscript; Risa D. Sargent contributed critically to the revisions of the drafts.

## CONFLICT OF INTEREST STATEMENT

The authors have no conflicts of interest to declare.

## STATEMENT ON INCLUSION

We are based in the region/country where the study was carried out (Vancouver, Canada). We worked directly with land planners and managers (Vancouver Parks Board) to ensure that our research outcomes help to address local management needs and questions.

## Supporting information


**Table S1.** Site details.
**Table S2.** Prior distributions for parameters estimated by the abundance GLMM.
**Table S3.** Prior distributions for parameters estimated by the binmix model.
**Table S4.** Prior distributions for parameters estimated by the multimix model.
**Figure S1.** We conducted abundance surveys at ten urban park sites in Vancouver, Canada.
**Figure S2.** We targeted 8 wild species in our abundance surveys: *Agapostemon texanus* (a), *Andrena prunorum* (b), *Anthidium oblongatum* (c), *Bombus mixtus* (d), *Bombus flavifrons* (e), *Halictus rubicundus* (f), native *Megachile* spp. (g), and *Melissodes microstictus* (h).
**Figure S3.** Mark‐recapture method.
**Figure S4.** Posterior predictive check plots for GLMM (a), binmix model (b), and multimix model (c), and for models using a Poisson distribution for abundance which showed poor fit (d–f).
**Figure S5.** Traceplots for the GLMM (a), binmix model (b), and multimix model (c).
**Figure S6.** Pairs plots for the GLMM (a), binmix model (b), and multimix model (c).
**Figure S7.** Expected increases in wild bee abundance in restored sites based on field data using GLMM, binmix, or multimix models (a); and change in increased expected abundance as bias for the restoration effect estimate increases (b).

## Data Availability

All data and code are available from the Figshare Digital Repository https://figshare.com/projects/Estimating_the_ecological_drivers_of_insect_abundance_when_detection_is_imperfect/264136 (Ulrich & Sargent, 2025a).
