# Supplementary material for: Estimating the ecological drivers of insect abundance when detection is imperfect
Source: J Anim Ecol. 2025 Oct 22;94(12):2641–54. doi: 10.1111/1365-2656.70159 (PMC12673238; doi:10.1111/1365-2656.70159)
Supplement: Supplementary file 1 — Table S1. Site details. Table S2. Prior distributions for parameters estimated by the abundance GLMM. Table S3. Prior distributions for parameters estimated by the binmix model. Table S4. Prior distributions for parameters estimated by the multimix model. Figure S1. We conducted abundance surveys at ten urban park sites in Vancouver, Canada. Figure S2. We targeted 8 wild species in our abundance surveys: Agapostemon texanus (a), Andrena prunorum (b), Anthidium oblongatum (c), Bombus mixtus (d), Bombus flavifrons (e), Halictus rubicundus (f), native Megachile spp. (g), and Melissodes microstictus (h). Figure S3. Mark‐recapture method. Figure S4. Posterior predictive check plots for GLMM (a), binmix model (b), and multimix model (c), and for models using a Poisson distribution for abundance which showed poor fit (d–f). Figure S5. Traceplots for the GLMM (a), binmix model (b), and multimix model (c). Figure S6. Pairs plots for the GLMM (a), binmix model (b), and multimix model (c). Figure S7. Expected increases in wild bee abundance in restored sites based on field data using GLMM, binmix, or multimix models (a); and change in increased expected abundance as bias for the restoration effect estimate increases (b). [file JANE-94-2641-s001.docx]

**Supporting Information** to accompany “Estimating the ecological drivers of insect abundance when detection is imperfect”.

**Authors**: Jens Ulrich, Risa D. Sargent

**Supplemental Tables**

**Table S1: Site Details.** We conducted wild bee abundance surveys in 1-hectare plots located in ten urban park sites in Vancouver, Canada. Detailed vegetation and site management data were reported by Ulrich and Sargent 2025.

| **Site name** | **Treatment group** | **Latitude** | **Longitude** |
| --- | --- | --- | --- |
| Killarney Park | Restored | 49.227468 | -123.046616 |
| Locarno Park | Restored | 49.273942 | -123.206008 |
| Oak Meadows Park | Restored | 49.238607 | -123.126438 |
| Prince of Wales Park | Restored | 49.243958 | -123.158084 |
| West Memorial Park | Restored | 49.243227 | -123.189281 |
| Bobolink Park | Control | 49.21503 | -123.057 |
| Gordon Park | Control | 49.223993 | -123.069652 |
| Kensington Park | Control | 49.23772 | -123.076 |
| Moberly Park | Control | 49.215687 | -123.086136 |
| Quilchena Park | Control | 49.242885 | -123.148097 |

**Table S2: Prior distributions for parameters estimated by the abundance GLMM.**

| **Parameter** | **Parameter meaning** | **Prior Distribution** | **Justification** |
| --- | --- | --- | --- |
| $\alpha_{0}$ | Global intercept | $Normal$(0, 2) | Weakly-informative centered prior. |
| $\alpha_{1}\left[ species \right]$ | Species-specific intercept effects | $Normal$(0, $\sigma_{\alpha_{1}}$) | Weakly-informative centered prior. |
| $\sigma_{\alpha_{1}}$ | Standard deviation of species-specific intercept effects | $Half-Normal$(0, 2) | Weakly-informative prior placing more weight near zero, i.e., small variation among groups (positive values only). |
| $\alpha_{2}\left[ site \right]$ | Site-specific intercept effects | $Normal$(0, $\sigma_{\alpha_{2}}$) | Weakly-informative centered prior. |
| $\sigma_{\alpha_{2}}$ | Variation in site-specific intercept effects | $Half-Normal$(0, 1) | Weakly-informative prior placing more weight near zero, i.e., small variation among groups (positive values only). |
| $\alpha_{3}\left[ year \right]$ | Year intercept effects | $Normal$(0, 1) | Weakly-informative centered prior. |
| $\alpha_{4}\left[ species \right]$ | Species-specific effects of restoration | $Normal$(0, $\sigma_{\alpha_{4}}$) | Weakly-informative centered prior. |
| $\sigma_{\alpha_{4}}$ | Variation in species-specific effects of restoration | $Half-Normal$(0, 2) | Weakly-informative prior placing more weight near zero, i.e., small variation among groups (positive values only). |
| $\phi$ | Negative binomial dispersion parameter | $Half-Normal$(0, 1) | Weakly-informative prior placing more weight near zero, i.e., overdispersion in counts (positive values only). |

**Table S3: Prior distributions for parameters estimated by the binmix model.**

| **Parameter** | **Parameter meaning** | **Prior Distribution** | **Justification** |
| --- | --- | --- | --- |
| *ABUNDANCE PARAMETERS* | | | |
| $\alpha_{0}$ | Global intercept | $Normal$(0, 2) | Weakly-informative centered prior. |
| $\alpha_{1}\left[ species \right]$ | Species-specific intercept effects | $Normal$(0, $\sigma_{\alpha_{1}}$) | Weakly-informative centered prior. |
| $\sigma_{\alpha_{1}}$ | Standard deviation of species-specific intercept effects | $Half-Normal$(0, 2) | Weakly-informative prior placing more weight near zero, i.e., small variation among groups (positive values only). |
| $\alpha_{2}\left[ site \right]$ | Site-specific intercept effects | $Normal$(0, $\sigma_{\alpha_{2}}$) | Weakly-informative centered prior. |
| $\sigma_{\alpha_{2}}$ | Variation in site-specific intercept effects | $Half-Normal$(0, 1) | Weakly-informative prior placing more weight near zero, i.e., small variation among groups (positive values only). |
| $\alpha_{3}\left[ year \right]$ | Year intercept effects | $Normal$(0, 1) | Weakly-informative centered prior. |
| $\alpha_{4}\left[ species \right]$ | Species-specific effects of restoration | $Normal$(0, $\sigma_{\alpha_{4}}$) | Weakly-informative centered prior. |
| $\sigma_{\alpha_{4}}$ | Variation in species-specific effects of restoration | $Half-Normal$(0, 2) | Weakly-informative prior placing more weight near zero, i.e., small variation among groups (positive values only). |
| $\phi$ | Negative binomial dispersion parameter | $Half-Normal$(0, 1) | Weakly-informative prior placing more weight near zero, i.e., overdispersion in counts (positive values only). |
| *ABUNDANCE PARAMETERS* | | | |
| $\beta_{0}$ | Global intercept | $Normal$(0, 2) | Weakly-informative centered prior. |
| $\beta_{1}\left[ species \right]$ | Species-specific intercept effects | $Normal$(0, $\sigma_{\beta_{1}}$) | Weakly-informative centered prior. |
| $\sigma_{\beta_{1}}$ | Variation in species-specific intercept effects | $Half-Normal$(0, 2) | Weakly-informative prior placing more weight near zero, i.e., small variation among groups (positive values only). |
| $\beta_{2}\left[ site \right]$ | Site-specific intercept effects | $Normal$(0, $\sigma_{\beta_{2}}$) | Weakly-informative centered prior. |
| $\sigma_{\beta_{2}}$ | Variation in site-specific intercept effects | $Half-Normal$(0, 1) | Weakly-informative prior placing more weight near zero, i.e., small variation among groups (positive values only). |
| $\beta_{3}\left[ year \right]$ | Year intercept effects | $Normal$(0, 1) | Weakly-informative centered prior. |
| $\beta_{4}\left[ species \right]$ | Species-specific effects of restoration | $Normal$(0, $\sigma_{\beta_{4}}$) | Weakly-informative centered prior. |
| $\sigma_{\beta_{4}}$ | Variation in species-specific effects of restoration | $Half-Normal$(0, 2) | Weakly-informative prior placing more weight near zero, i.e., small variation among groups (positive values only). |

**Table S4: Prior distributions for parameters estimated by the multimix model.**

| **Parameter** | **Parameter meaning** | **Prior Distribution** | **Justification** |
| --- | --- | --- | --- |
| *ABUNDANCE PARAMETERS* | | | |
| $\alpha_{0}$ | Global intercept | $Normal$(0, 2) | Weakly-informative centered prior. |
| $\alpha_{1}\left[ species \right]$ | Species-specific intercept effects | $Normal$(0, $\sigma_{\alpha_{1}}$) | Weakly-informative centered prior. |
| $\sigma_{\alpha_{1}}$ | Standard deviation of species-specific intercept effects | $Half-Normal$(0, 2) | Weakly-informative prior placing more weight near zero, i.e., small variation among groups (positive values only). |
| $\alpha_{2}\left[ site \right]$ | Site-specific intercept effects | $Normal$(0, $\sigma_{\alpha_{2}}$) | Weakly-informative centered prior. |
| $\sigma_{\alpha_{2}}$ | Variation in site-specific intercept effects | $Half-Normal$(0, 1) | Weakly-informative prior placing more weight near zero, i.e., small variation among groups (positive values only). |
| $\alpha_{3}\left[ year \right]$ | Year intercept effects | $Normal$(0, 1) | Weakly-informative centered prior. |
| $\alpha_{4}\left[ species \right]$ | Species-specific effects of restoration | $Normal$(0, $\sigma_{\alpha_{4}}$) | Weakly-informative centered prior. |
| $\sigma_{\alpha_{4}}$ | Variation in species-specific effects of restoration | $Half-Normal$(0, 2) | Weakly-informative prior placing more weight near zero, i.e., small variation among groups (positive values only). |
| $\phi$ | Negative binomial dispersion parameter | $Half-Normal$(0, 1) | Weakly-informative prior placing more weight near zero, i.e., overdispersion in counts (positive values only). |
| *ABUNDANCE PARAMETERS* | | | |
| $\beta_{0}$ | Global intercept | $Normal$(0, 2) | Weakly-informative centered prior. |
| $\beta_{1}\left[ species \right]$ | Species-specific intercept effects | $Normal$(0, $\sigma_{\beta_{1}}$) | Weakly-informative centered prior. |
| $\sigma_{\beta_{1}}$ | Variation in species-specific intercept effects | $Half-Normal$(0, 2) | Weakly-informative prior placing more weight near zero, i.e., small variation among groups (positive values only). |
| $\beta_{2}\left[ site \right]$ | Site-specific intercept effects | $Normal$(0, $\sigma_{\beta_{2}}$) | Weakly-informative centered prior. |
| $\sigma_{\beta_{2}}$ | Variation in site-specific intercept effects | $Half-Normal$(0, 1) | Weakly-informative prior placing more weight near zero, i.e., small variation among groups (positive values only). |
| $\beta_{3}\left[ year \right]$ | Year intercept effects | $Normal$(0, 1) | Weakly-informative centered prior. |
| $\beta_{4}\left[ species \right]$ | Species-specific effects of restoration | $Normal$(0, $\sigma_{\beta_{4}}$) | Weakly-informative centered prior. |
| $\sigma_{\beta_{4}}$ | Variation in species-specific effects of restoration | $Half-Normal$(0, 2) | Weakly-informative prior placing more weight near zero, i.e., small variation among groups (positive values only). |

**Supplemental Figures**


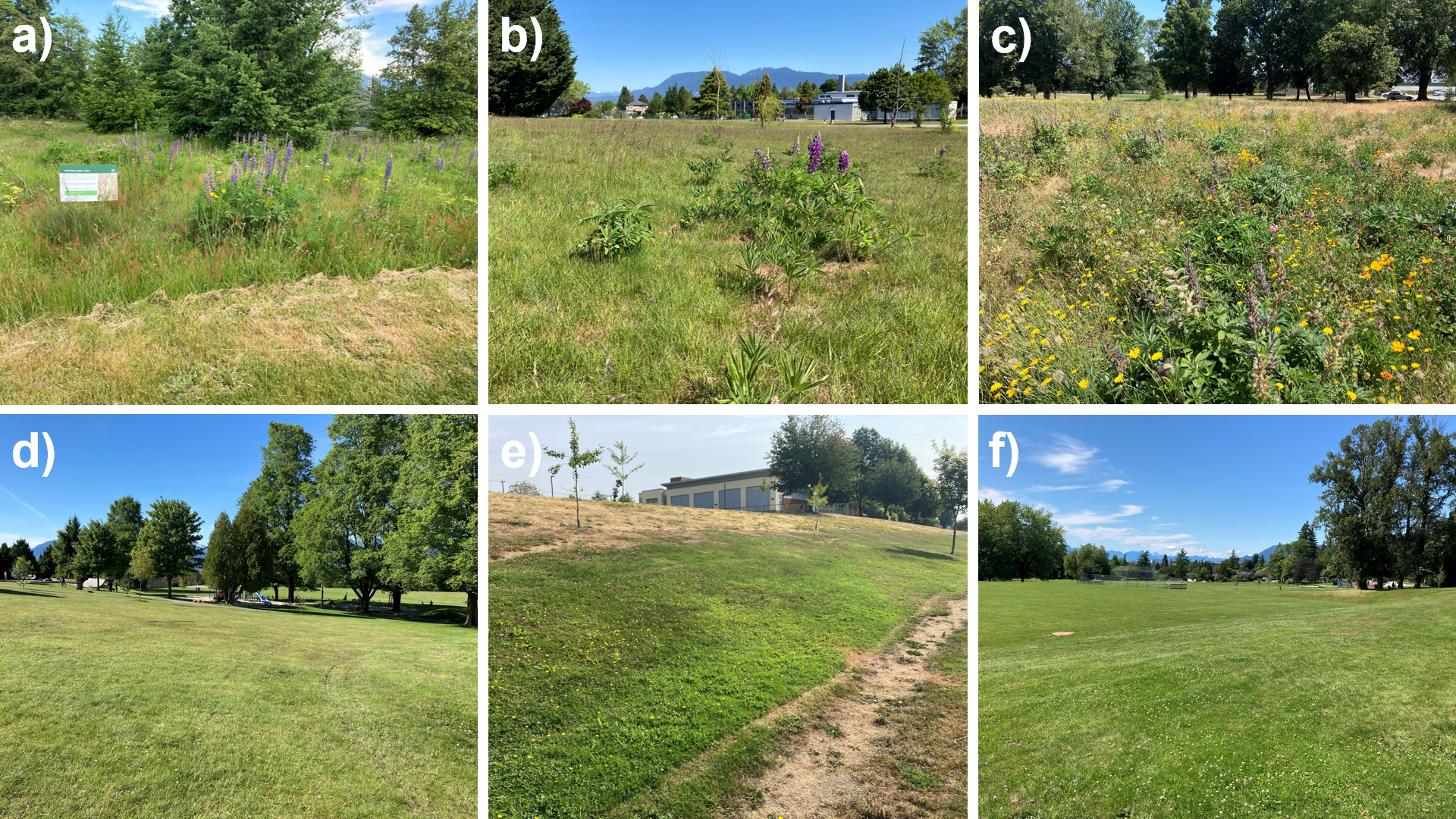


**Figure S1: We conducted abundance surveys at ten urban park sites in Vancouver, Canada.** Five of the sites were historically turfgrass dominated but received a restoration amendment of about 1 hectare area. The restoration consisted of herbaceous flower seeding combined with no mowing during the spring and summer months. Some restored site examples include Oak Meadows Park (a), Prince of Wales Park (b), and Killarney Park (c). In control sites, turfgrass areas were not amended with a restoration. Some control site examples include Kensington Park (d), Moberly Park (e), and Quilchena Park (f). Restored sites had significantly more flower resources and were characterized by taller herbaceous vegetation.


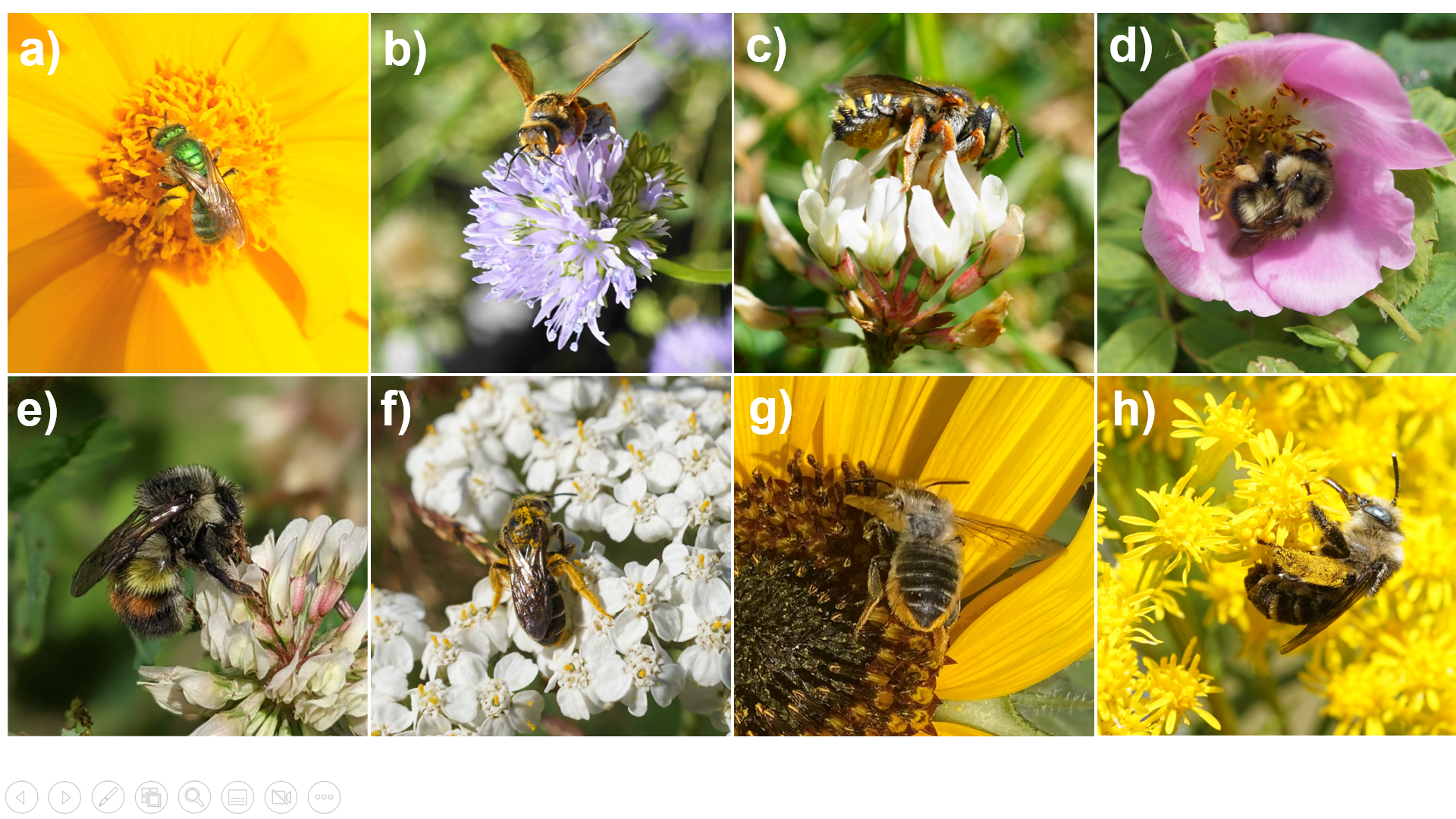


**Figure S2: We targeted 8 wild species in our abundance surveys:** *Agapostemon texanus* (a), *Andrena prunorum* (b), *Anthidium oblongatum* (c), *Bombus mixtus* (d), *Bombus flavifrons* (e), *Halictus rubicundus* (f), native *Megachile spp.* (g), and *Melissodes microstictus* (h). The picture of *B. flavifrons* was taken by Sarah Knoerr and used with permission.


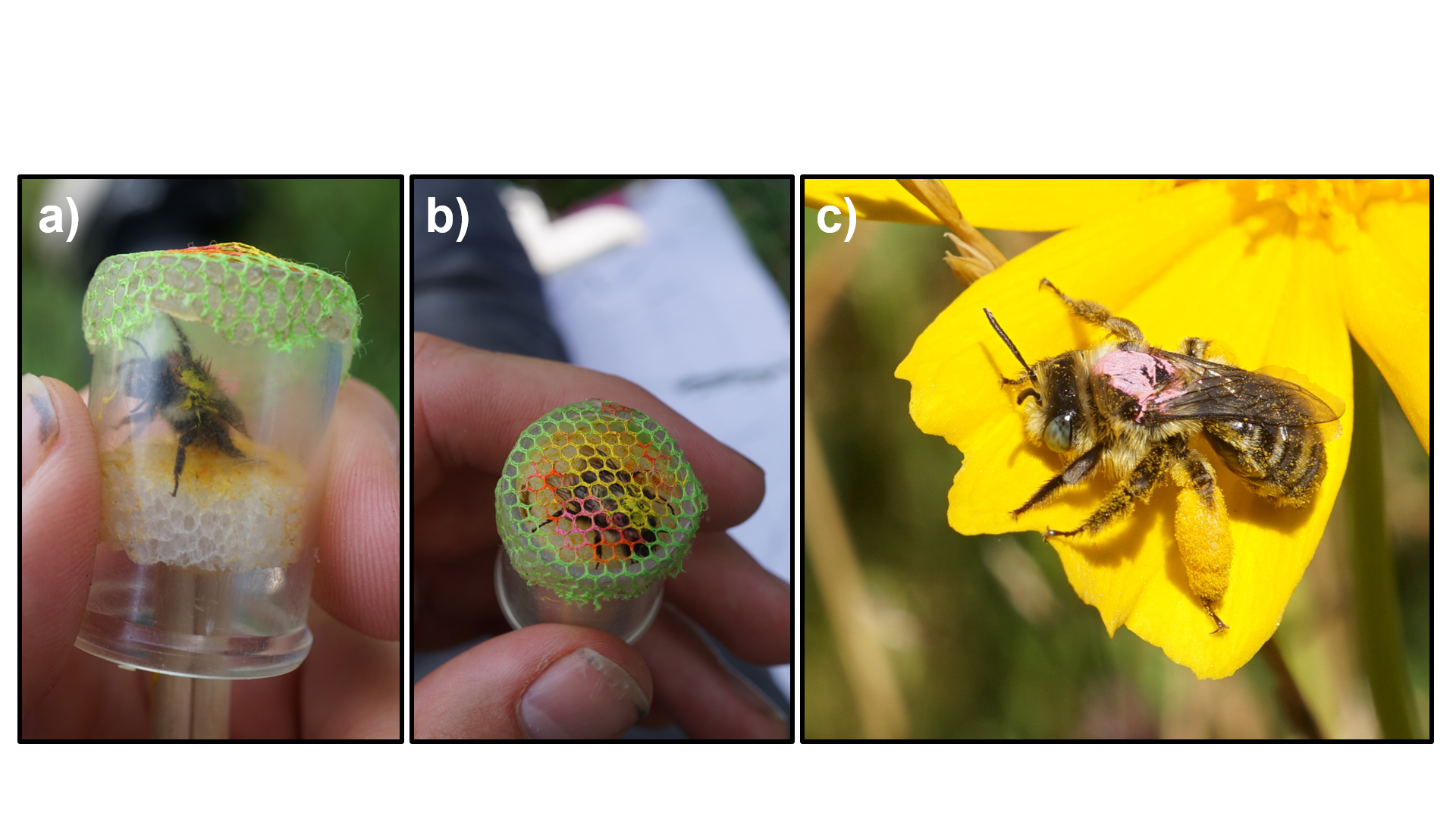


**Figure S3: Mark-recapture method.** We placed chilled bees in a modified vial with a styrofoam plunger on one end (a). Chilling the bees allowed for easier handling. We used the plunger to press the thorax of the bee against a mesh covering on the opposite side of the vial (b). We used a Molotow brand paint pen to place a mark on the thorax. The mark was colour-coded to correspond to the unique capture date. In (b), the bee was captured on two occasions. It has a yellow mark indicating the first capture and a pink mark representing the second. After marking, we released the bees back to the survey area. We observed that bees then resumed normal foraging behaviour, usually after sunning themselves for a few minutes (c).

1.
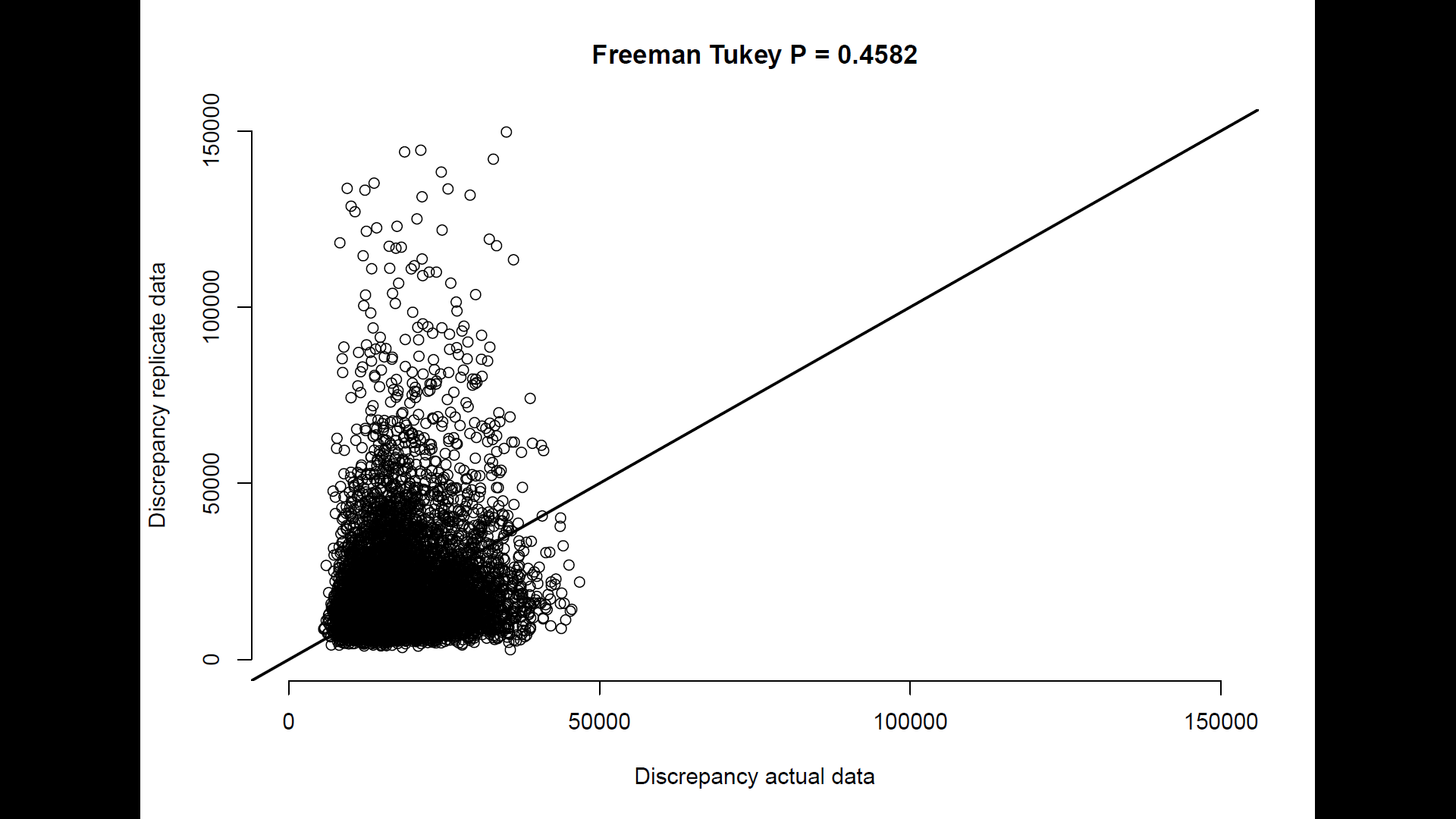

2.
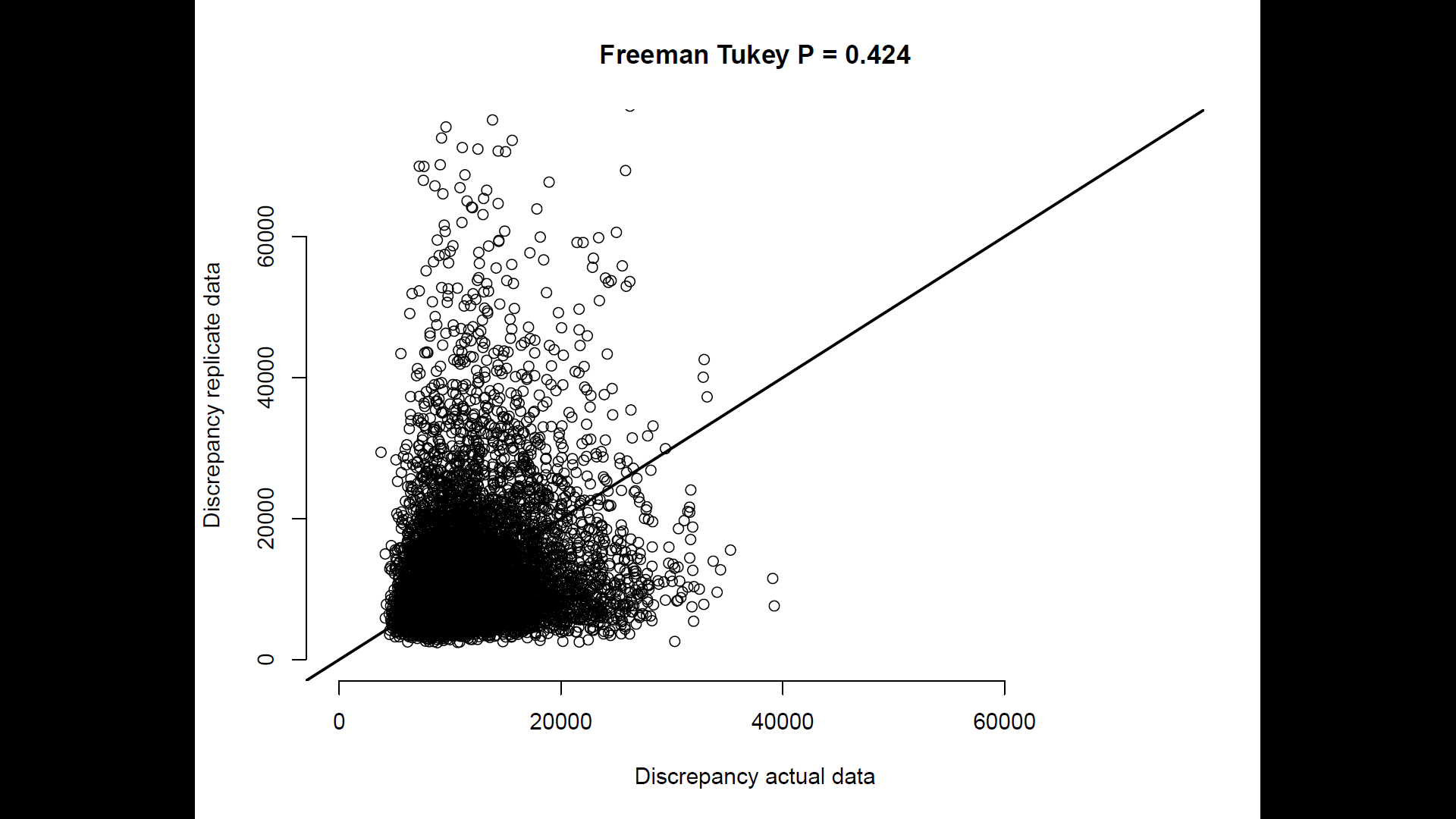

3.
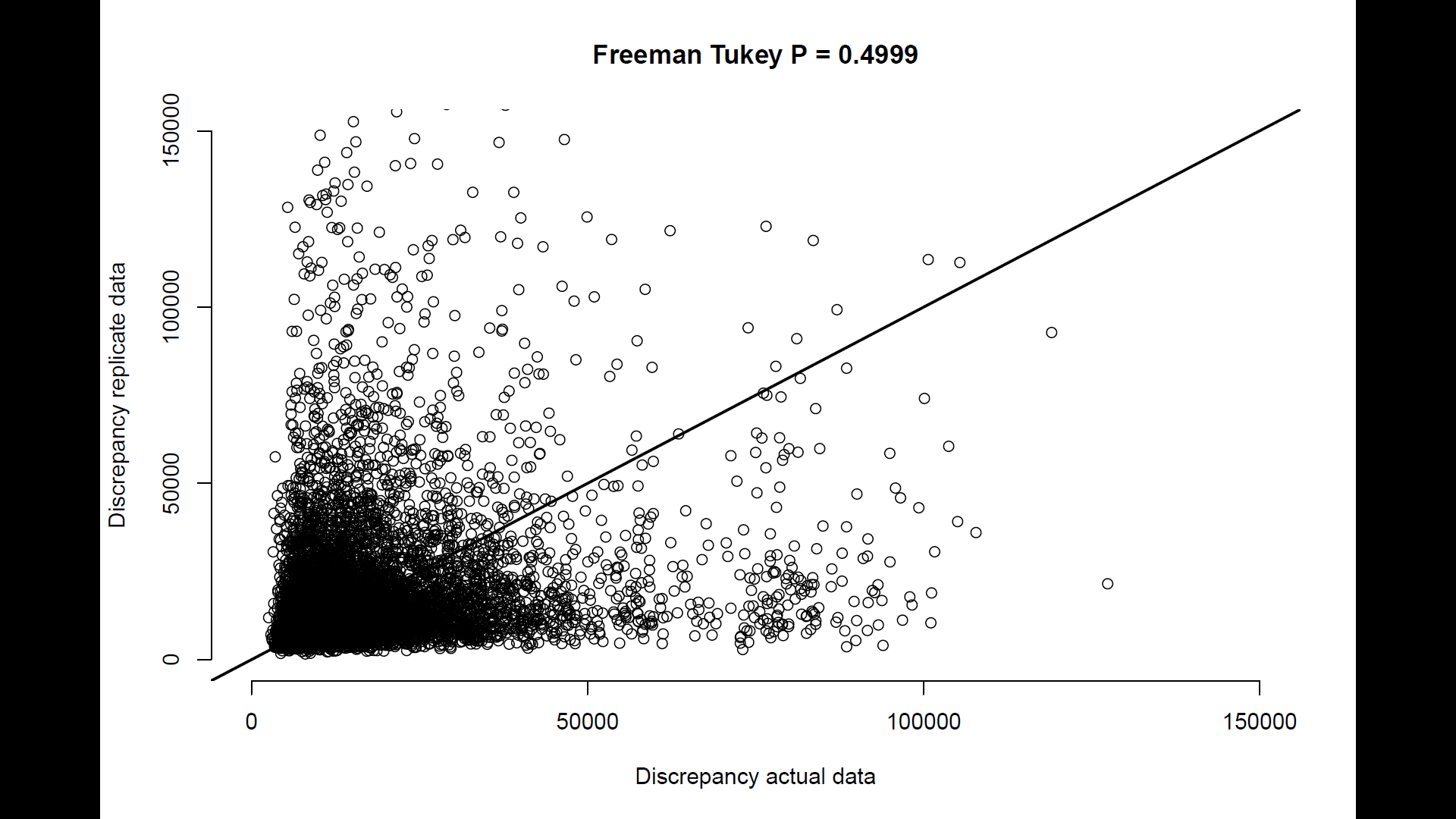

4.
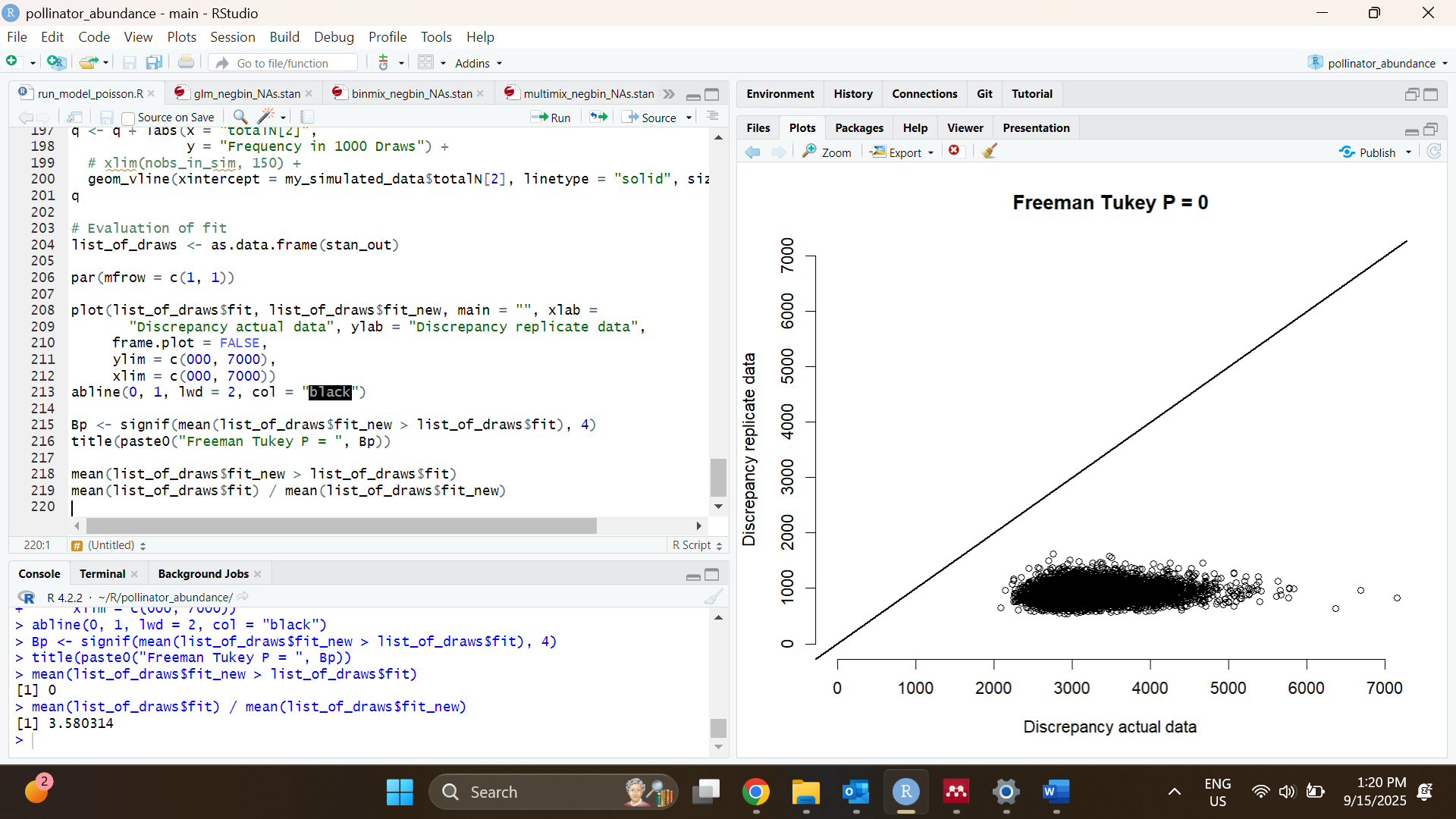

5.
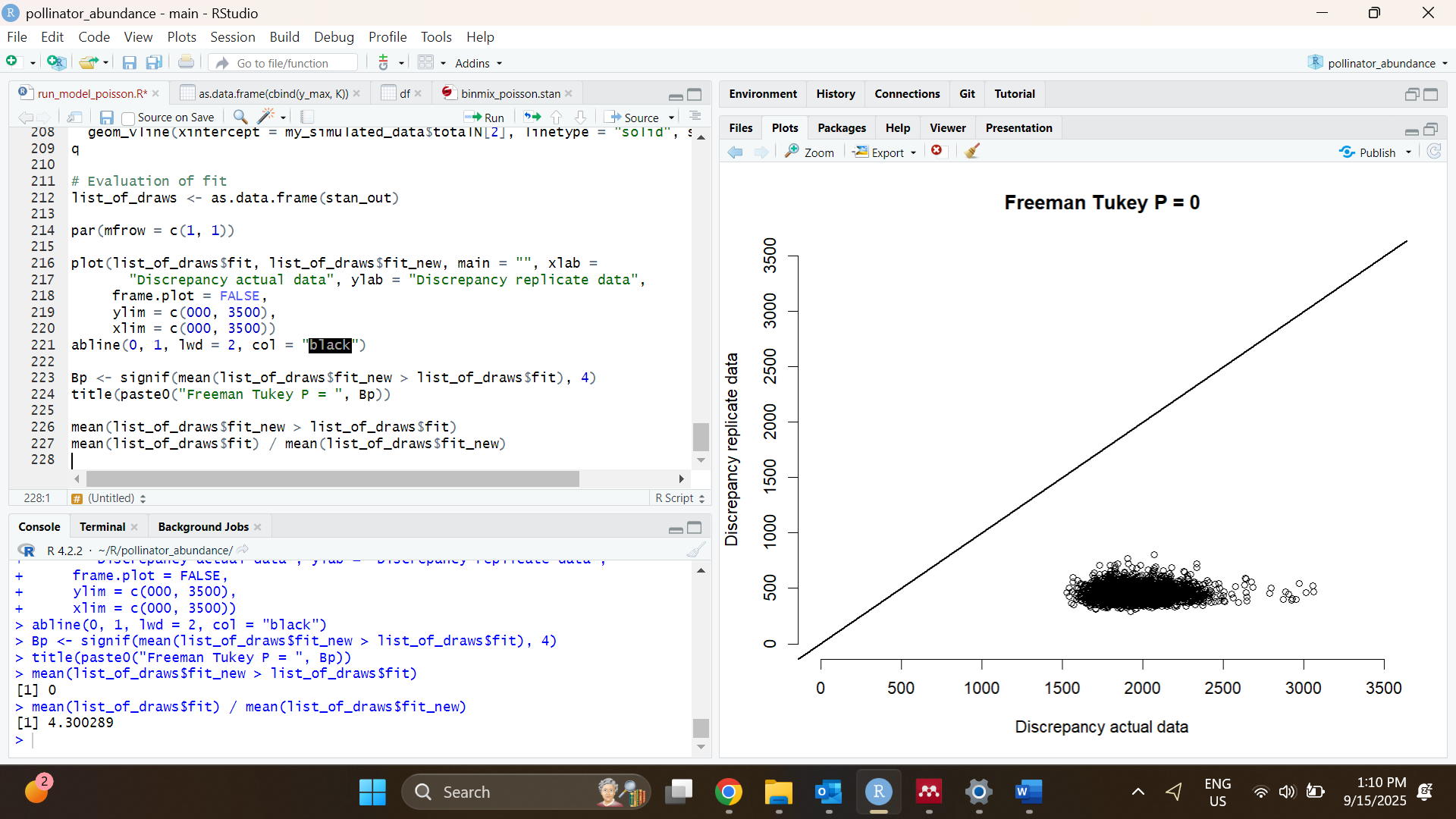

6.
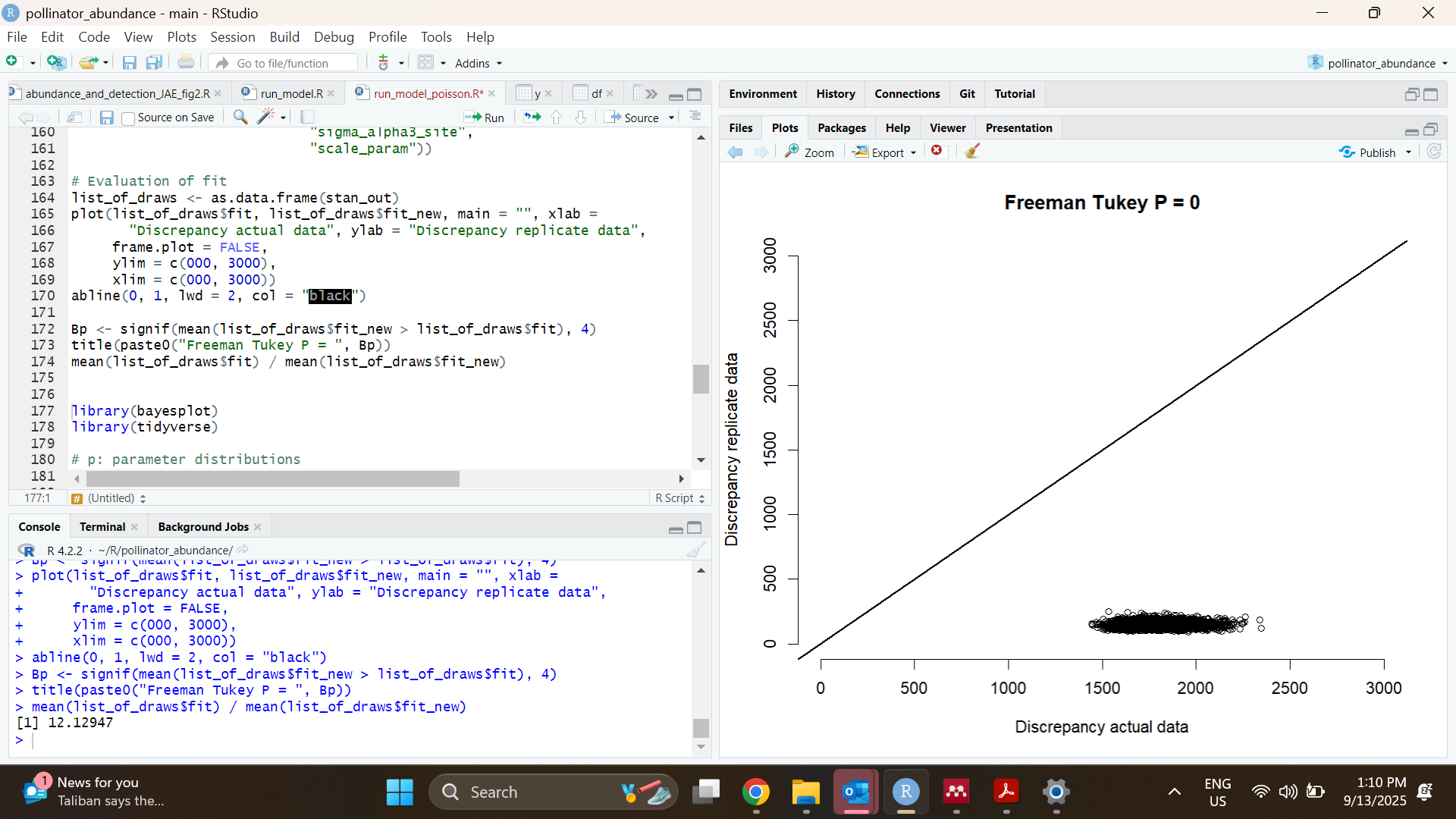


**Figure S4: Posterior predictive check plots for GLMM (a), binmix model (b), and multimix model (c), and for models using a Poisson distribution for abundance which showed poor fit (d, e, and f).** To assess model fit, we conducted Freeman-Tukey statistic discrepancy tests for each model (Conn et al., 2018; Kery and Royle, 2020). This test evaluates the difference in discrepancy between simulated data or real data versus an expected outcome. To conduct the test, we simulated some data (y_replicated) using the parameter estimates at each step of the HMC run. Data were counts of the number of individuals detected per species per site-survey (a, b) or the number of individuals detected at least once per species per site across all three surveys (c). We then simulated some new data from the same parameter estimates (y_new). We calculated the Freeman-Tukey discrepancy for all y_new across all sampling events *i*, and also for our actual data y:

$Fit discrepancy(y_{\mathrm{new}})$ = $\sum_{i}^{R} (square(y\_new[i] - y\_replicated [i]) / (y\_replicated [i] + 0.5)$

$Fit discrepancy(y)$ = $\sum_{i}^{R} (square(y[i] - y\_replicated [i]) / (y\_replicated [i] + 0.5)$

We then calculated the frequency in the posterior distribution of samples at which the fit discrepancy for $y_{\mathrm{new}}$ is greater than the fit discrepancy for $y$. Values near 0.5 suggest reasonable goodness of fit, i.e., that newly simulated data tend to be more or less as far away from some other simulated data as our real data. Values below 0.05 or greater than 0.95 indicate critical lack of model fit. FTP values were less than 0.05 for models for our field data that used a Poisson distribution for the abundance random variable, which prompted us to use negative binomial distributions.

Conn, P. B., Johnson, D. S., Williams, P. J., Melin, S. R., & Hooten, M. B. (2018). A guide to Bayesian model checking for ecologists. Ecological Monographs, 88(4), 526-542.

Kéry, M., & Royle, J. A. (2020). Applied hierarchical modeling in ecology: Analysis of distribution, abundance and species richness in R and BUGS: Volume 2: Dynamic and advanced models. Academic Press.

1.
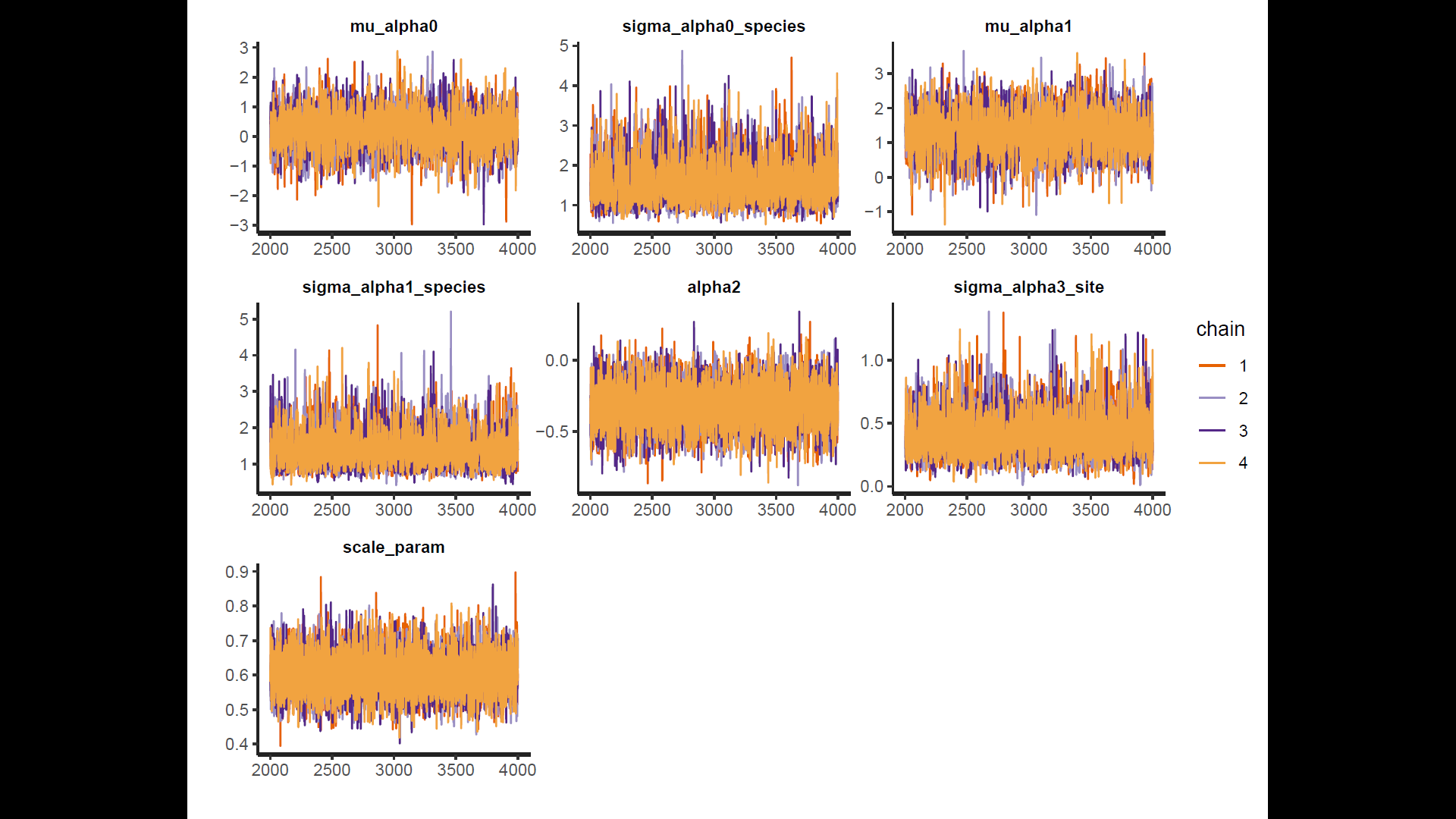

2.
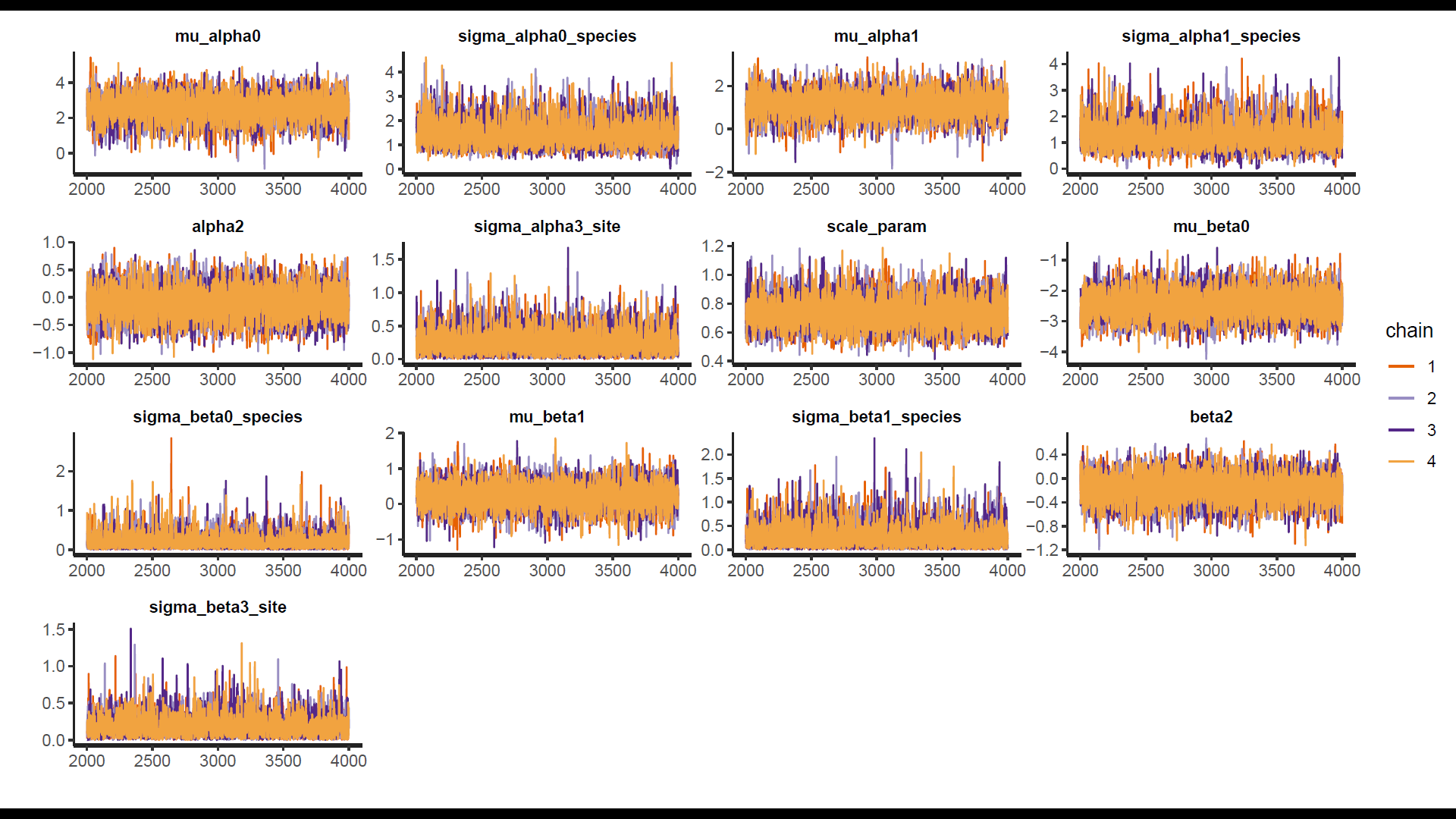

3.
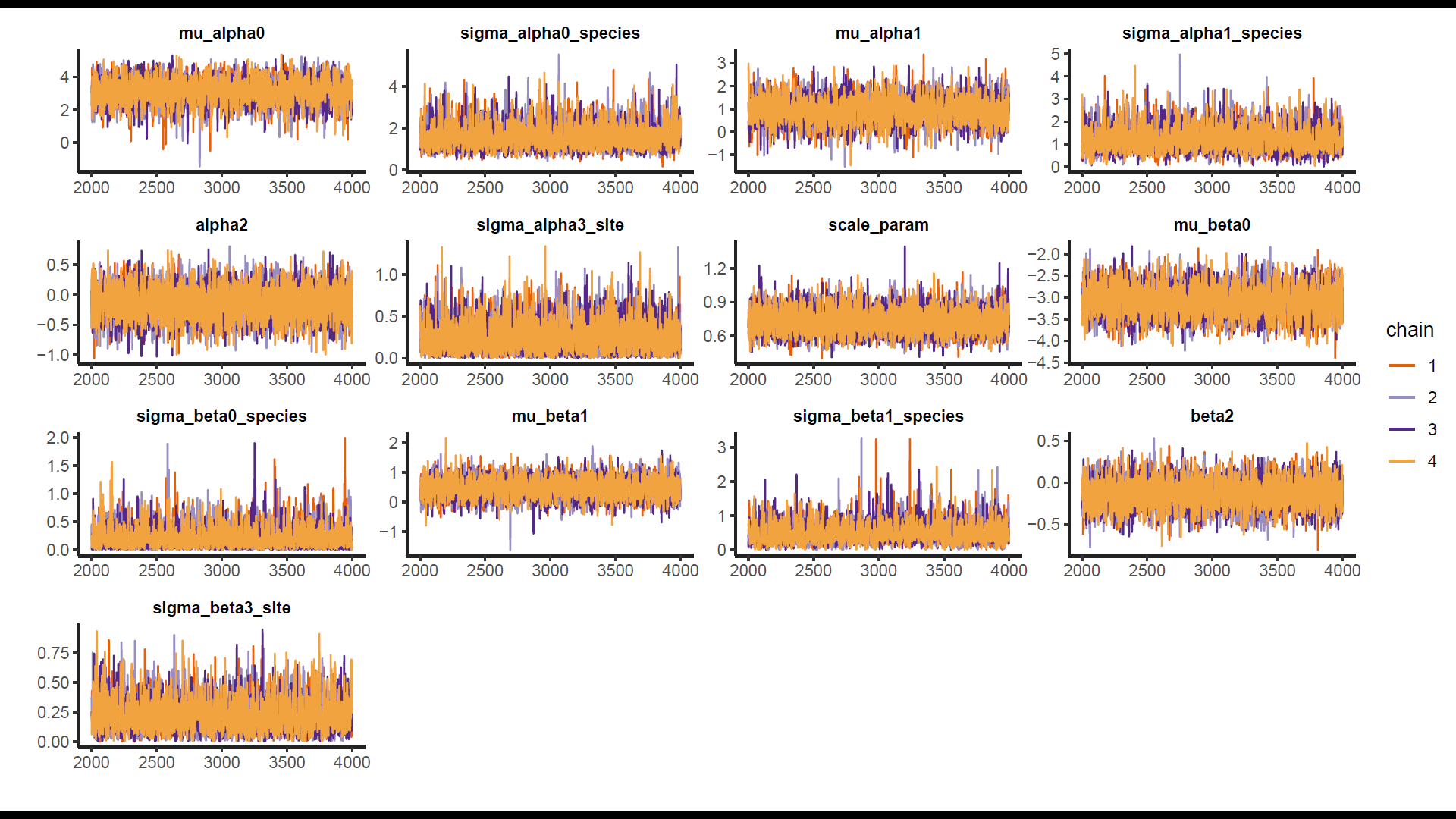


**Figure S5: Traceplots for the GLMM (a), binmix model (b), and multimix model (c).** Overlapping HMC traces indicate that all 4 chains have converged on a similar parameter space.

1.
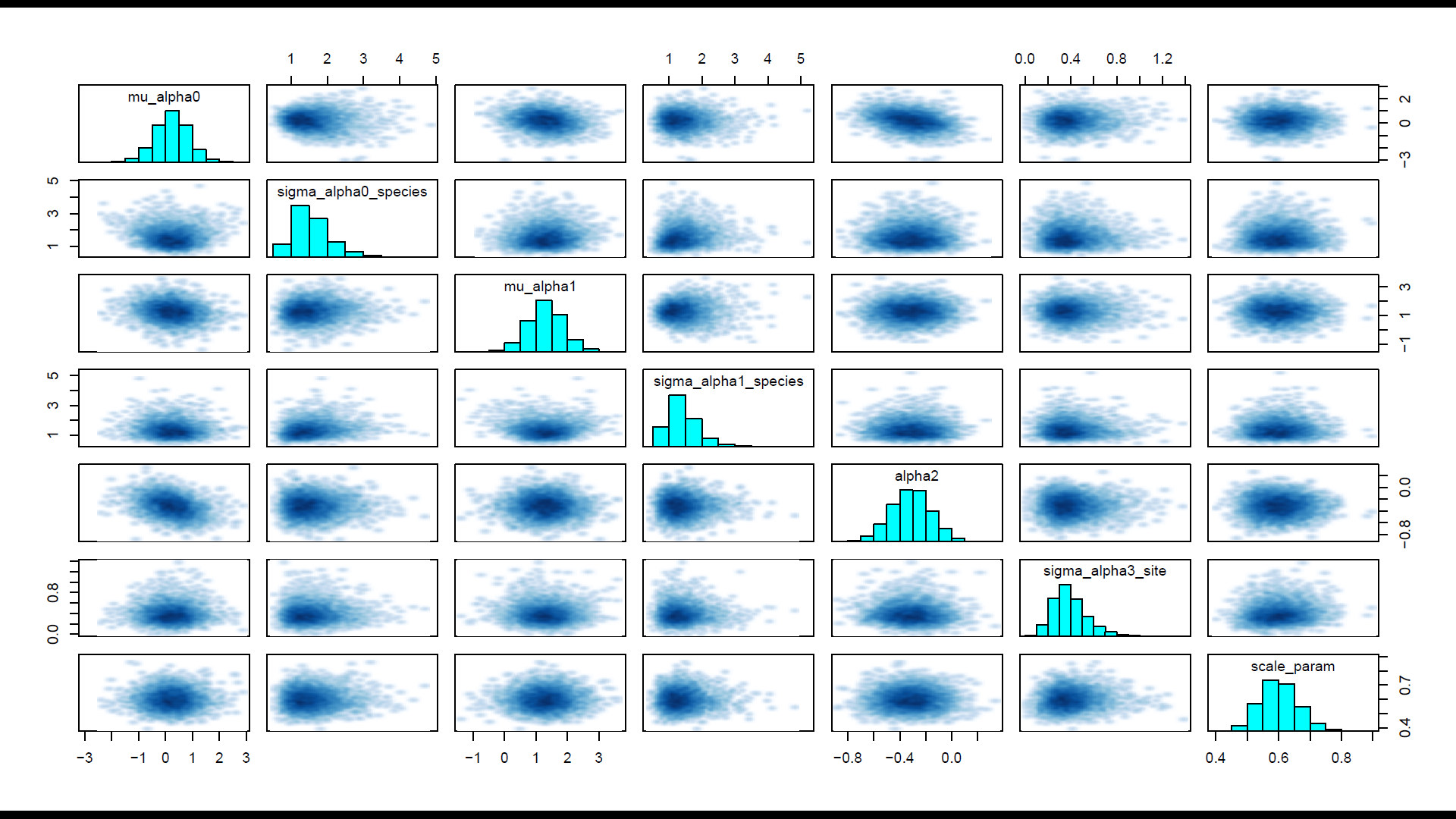

2.
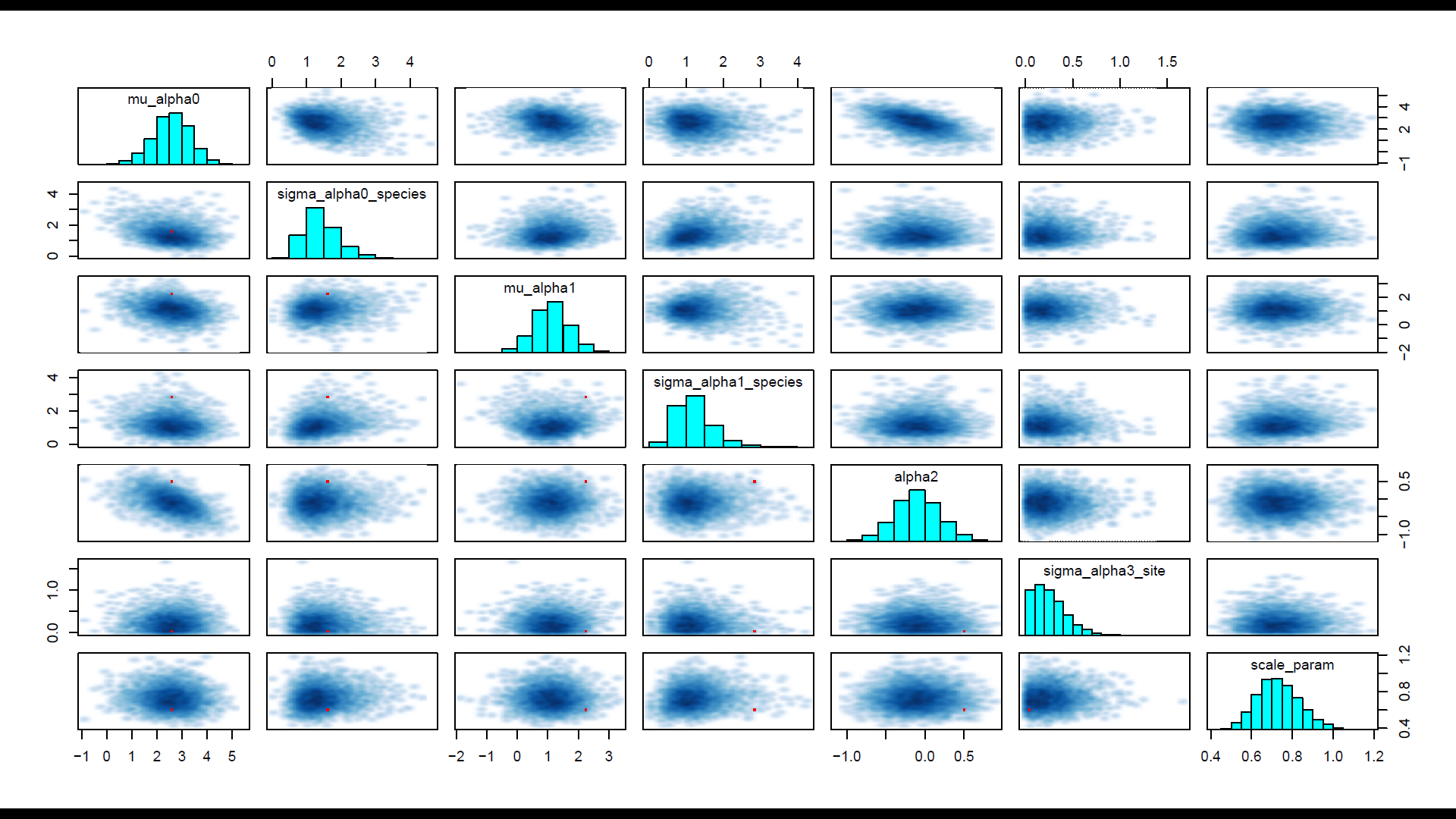

3.
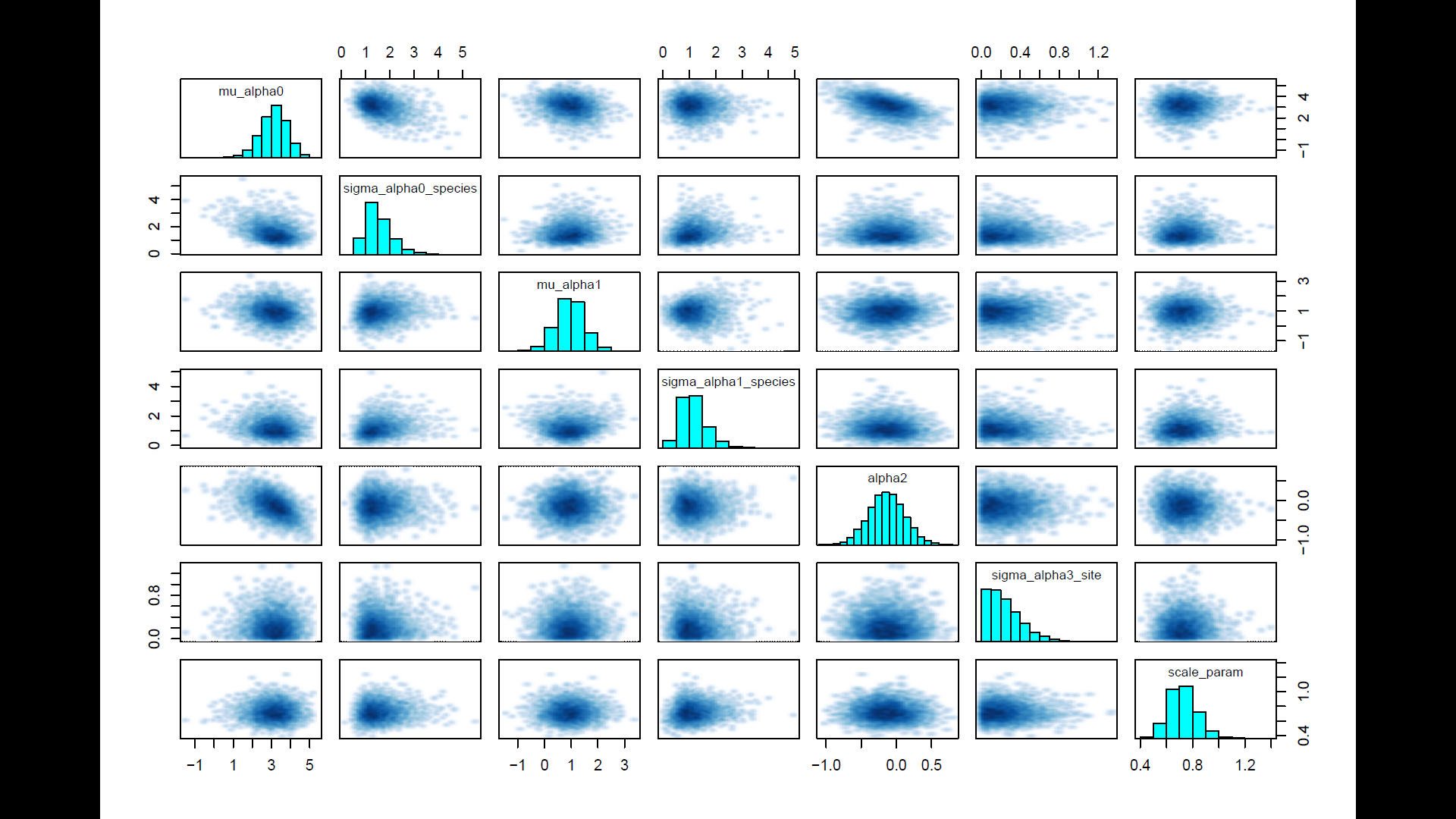


**Figure S6: Pairs plots for the GLMM (a), binmix model (b), and multimix model (c).** Pairs plot are shown for a sample subset of parameters (abundance parameters). The lack of divergent transitions and no visual barriers in the parameter space in these pairs plots search support that the posterior distribution has been fully explored.


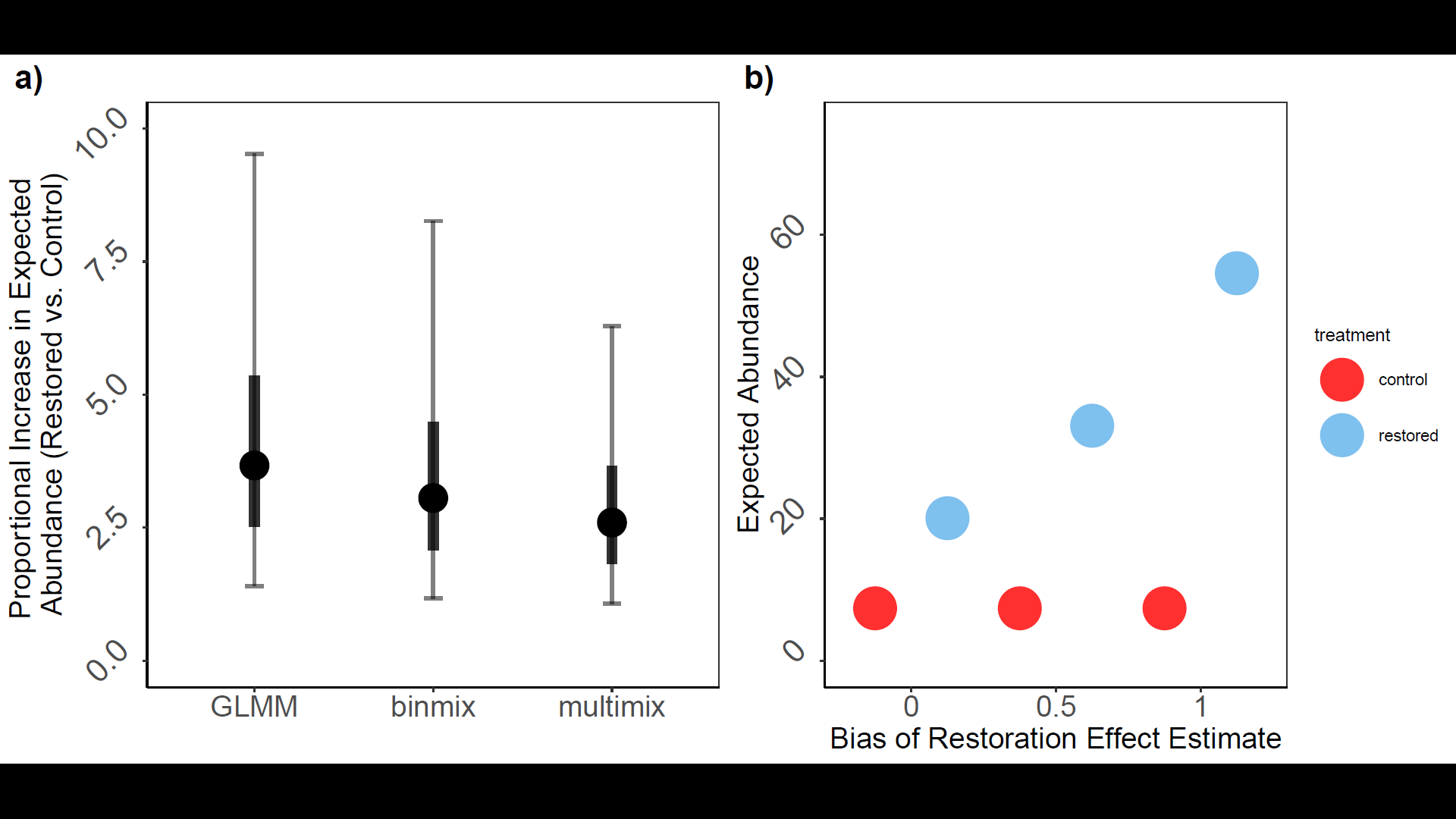


**Figure S7: Expected increases in wild bee abundance in restored sites based on field data using GLMM, binmix, or multimix models (a); and change in increased expected abundance as bias for the restoration effect estimate increases (b).** We calculated proportional increase in expected abundance (a) by dividing expected abundance for an average species at an average restored site by expected abundance for an average species at an average control site. Expected abundance at control sites was calculated as *exp*(intercept), while the expected abundance at restored sites was calculated as *exp*(intercept + estimated effect of restoration on abundance). The mean and 50% and 90% Bayesian credible intervals are shown. To demonstrate the effects of estimate bias on biological interpretation of an environmental driver of abundance we show the expected abundance in control and restored sites under different amounts of bias. We used our simulation settings with an abundance intercept of 2, and an effect of habitat restoration of 1. The expected abundance in a control site is *exp*(2) =~ 7. When bias = 0 (model estimates effect of restoration with high accuracy) the expected abundance in a restored site is *exp*(2 + 1) =~ 20. When bias = 0.5 (model overestimates effect of restoration by 0.5) the expected abundance in a restored site is *exp*(2 + 1+ 0.5) =~ 33. In this scenario, the biased model would overestimate increases by about 100% (increase of 26 individuals rather than true increase of 13). When bias = 1 (model overestimates effect of restoration by 1) the expected abundance in a restored site is *exp*(2 + 1+ 1) =~ 55. In this scenario, the biased model would overestimate increases by about 270% (increase of 48 individuals rather than true increase of 13).
